# Determination of depth and field size dependence of multileaf collimator transmission in intensity‐modulated radiation therapy beams

**DOI:** 10.1120/jacmp.v8i4.2693

**Published:** 2007-10-24

**Authors:** Piotr Zygmanski, Florin Rosca, Dnyanesh Kadam, Friedlieb Lorenz, Adrian Nalichowski, Laurence Court, Lee Chin

**Affiliations:** ^1^ Department of Radiation Oncology Brigham and Women's Hospital and Harvard Medical School Boston Massachusetts U.S.A.

**Keywords:** dynamic MLC, MLC transmission, dosimetry, IMRT, closed MLC

## Abstract

Intensity‐modulated radiation therapy (IMRT) plans for the treatment of large and complex volumes may contain a relatively large contribution from multileaf collimator (MLC) transmission. In such cases, comprehensive characterization of direct and scatter MLC transmission is important. We designed a set of tests (open beam, closed static MLC, and dynamic MLC gap) to determine dosimetric MLC properties as a function of field size and depth at the central axis.

We developed a generalized model of MLC transmission to account for direct MLC transmission, MLC scatter, beam hardening, and leaf‐end transmission (dosimetric gap). The model is consistent with the beam model used in IMRT optimization. We tested the model for extreme asymmetric fields relevant for large targets and for split IMRT fields. We applied our MLC scatter estimation formula to clinically relevant cases and showed that MLC scatter is contributing an undesired background dose. This contribution is relatively large, especially in low‐dose regions. (For instance, a uniform extra dose may dramatically increase normal‐lung toxicity in thorax treatment.) For complex IMRT of large‐volume targets, we found direct MLC transmission dose to be as high as 30%, and MLC scatter, up to 10% within the target volume for the selected cases. We identified that the dose discrepancies between the IMRT planning system [Eclipse (Varian Medical Systems, Palo Alto, CA)] and ionization chamber measurements (inside and outside of the field) are attributable to an inadequate model of MLC transmission in the planning system (constant‐value model).

In the present study, we measured MLC transmission properties for Varian 6EX (6 MV) and 21EXs (6 and 10 MV) linear accelerators; however, the experimental method and theoretical model are more general.

PACS number: 87.53.‐j

## I. INTRODUCTION

Planning for intensity‐modulated radiation therapy (IMRT) and quality assurance (QA) for whole‐pelvis (for example, prostate plus regional lymph nodes), head‐and‐neck, and especially mesothelioma treatments may be challenging because of the complexity of the target (large and difficult geometry, often with irregular and concave shapes) and the demanding nature of the optimization objectives (multiple targets with different dose levels, up to a dozen organs at risk). The result may be highly complex and noisy fluence patterns with low monitor unit (MU) efficiency and small average multileaf collimator (MLC) gap.

The MU efficiency of dynamically delivered IMRT fields can be defined as a ratio of the MUs needed to deliver an open beam and IMRT for the same field size, depth, and average dose per field. The MU efficiency for an open beam would be 100%. For a completely blocked field (closed MLC), it would be equal to the MLC transmission [T≈1.4% for a 6‐MV Varian 21EX (Varian Medical Systems, Palo Alto, CA) linear accelerator (LINAC)].

In our clinical practice, we have encountered a relatively large number of IMRT plans for which the MU efficiency is less than 20%. Notably, 20% efficiency corresponds to about 81% of the delivered radiation being attenuated and only about 19% being transmitted through MLC gaps. Low MU efficiency of this kind implies an increased contribution of MLC transmission (mid‐leaf, interleaf, and tongue‐and‐groove), MLC‐induced beam hardening, and MLC scatter to the total dose.

In addition, because the field sizes for large targets range from about 20×20 cm to 35×38 cm, and the target cannot be covered by the range of MLC motion (14‐cm limit on field width), fluence patterns are often split into two or three subfluences. For instance, IMRT with 7 – 9 fields may result in 12 – 27 split subfields in the delivery. Dose attributable to MLC scatter from split fields accumulates in the total plan in the same way for normal organs and the target alike, but the normal organs may be more sensitive to the extra dose.

Because of cumulative effects, calculation of dose for such fluence patterns is highly sensitive to any slight inadequacies of the dose calculation model and also to the uncertainties in the dosimetric MLC parameters in the IMRT planning system. An IMRT planning system that assumes a constant‐value model of MLC transmission may be inadequate for complex and large‐target IMRT, for which the field size and the depth dependence of MLC transmission both matter.

Dosimetric MLC properties have been studied in the context of dynamic IMRT delivery either by direct measurement and theoretical calculation, or by Monte Carlo simulation^(^ ^1^ ^–^ ^7^ ^)^. To the best of our knowledge, although the problem of MLC scatter and beam hardening in IMRT has been identified,^(^ ^3^ ^–^ ^5^ ^)^ it has not been completely characterized and resolved.

The published data on MLC scatter and beam hardening are based primarily on Monte Carlo studies.^(^ ^3^ ^,^ ^4^ ^)^ For closed MLC, the Monte Carlo data indicate that MLC scatter significantly depends on field size and that MLC scatter and beam hardening also depend on depth and energy. For IMRT fields with a high component of closed MLC as compared with open beam (for example, 80% closed MLC plus 20% open beam), these dependencies are similarly applicable. Even though MLC scatter data obtained by techniques other than Monte Carlo studies have been reported, the experimental method that leads to the determination of those data was not fully described.^(5)^

Even within the Monte Carlo beam model, proper characterization of MLC properties may be difficult, and extensive experimental calibration of the MLC parameters used in the Monte Carlo code is required.^(^ ^8^ ^,^ ^9^ ^)^ This calibration has to be performed separately for each physical combination of MLC and LINAC, indicating that Monte Carlo–derived MLC parameters can be used primarily for a Monte Carlo dose calculation engine, but that for another dose calculation engine (planning system), other sets of calibration tests may need to be carried out.

Recently, additional experimental data on MLC scatter and comparison of dose outputs for dynamic sweeping gaps were reported for two commercial planning systems [Eclipse and Pinnacle for 2300CD and 21EX (Varian Medical Systems)]^(10)^. These commercial planning systems do not model MLC scatter, and the associated errors can be significant^(10)^.

In the present report, we introduce a phenomenologic beam model that explicitly includes MLC scatter. We systematically measure MLC transmission as a function of field size, depth, and beam energy, and we derive the model parameters. We consider MLC beam hardening only to the extent that it affects MLC parameters, which depend on depth. We provide an approximate formula for estimation of MLC scatter. We test the model for extreme asymmetric fields relevant to large targets and for split IMRT fields.

The focus of this work are the 6‐ and 10‐MV photon energy beams from Varian 6EX (6 MV only) and 21EX (6 and 10 MV) LINACs equipped with Millenium MLCs (120 leaves, rounded leaf design).

## II. MATERIALS AND METHODS

### A. Dosimetric MLC parameters

We introduce a phenomenologic method to simultaneously determine dosimetric MLC parameters:

- direct MLC transmission *T*,
- MLC scatter correction β, and
- dosimetric gap ΔG=(2RFO+CMO),

where radiation field offset (RFO) is the offset of MLC positions attributable to penetration of radiation through the rounded leaf ends, and center mechanical offset (CMO) is a mechanical offset that can be adjusted by the user (typically set to 0).^(^ ^2^ ^,^ ^11^ ^)^ The net effect of the two offsets RFO and CMO is sometimes called “dosimetric gap” in the Eclipse treatment planning system (TPS).

Except for interleaf transmission, dose profiles of open beam (OB), closed MLC (cMLC), and dynamic MLC gap (dMLCgap) normalized to the central axis are similar. The dynamic MLC tests and analysis presented here were designed to determine the MLC parameters in such a way that interference from other effects (extrafocal radiation, interleaf MLC transmission, tongue‐and‐groove effect) is either null or negligible. In principle, direct MLC transmission and MLC scatter radiation include both primary radiation and head scatter. Both the primary and the head scatter radiation are attenuated by the MLC.

In our model, MLC parameters include neither head scatter nor phantom scatter, both of which are effectively canceled by determining the appropriate dose ratio (same field size and depth). Changes in beam spectrum attributable to the MLC are reflected in the depth dependence of *T* [*T*(*d*)] and *β* [*β* (*d*)]. The spatial dependency of the MLC transmission attributable to the tongue‐and‐groove MLC design and its effect on complex IMRT delivery will be discussed in more detail in a future report.

The OB–cMLC–dMLCgap tests apply to dynamic MLC field sizes from $c_{x}\times c_{y}\approx 3\times 3\;\text{cm}$ to $c_{x}\times c_{y}\approx 14\times 40\;\text{cm}$. However, because MLC scatter is negligible for field sizes smaller than $c_{x}\times c_{y}\approx 5\times 5\;\text{cm}$, and because dosimetric gap is practically independent of field size, use of a minimum field size of $c_{x}\times c_{y}\approx 5\times 5\;\text{cm}$ is sufficient. Because field size dependence is smooth, the largest field sizes we use are $c_{x}\times c_{y}\approx 12\times 28\;\text{cm}$ with a 30×30 cm Solid Water (Gammex rmi, Middleton, WI) phantom and 14×38 cm with a 30×60 cm Solid Water phantom.

### B. Dose ratios

We limit phenomenologic modeling of the experimental data to dose ratios: $R_{\text{cMLC}}$ and $R_{\text{dMLCgap}}$. These ratios express the relationship between doses in test conditions and doses under the reference conditions at the central axis and a source‐to‐surface distance of 100 cm. These ratios are derived from the measured doses $D_{\text{OB}},D_{\text{cMLC}}$, and $D_{\text{dMLCgap}}$ for OB, cMLC, and dMLCgap accordingly:

(1(A)) $$R_{cMLC}=\frac{D_{cMLC}(c_{x},c_{y},d)}{D_{OB}(c_{x},c_{y},d)}\;\;\text{and}$$

(1(B)) $$R_{dMLCgap}=\frac{D_{dMLCgap}(c_{x},c_{y},d)}{D_{OB}(c_{x},c_{y},d)}.$$

The doses are measured for the same field size and depth $\left(c_{x}\times c_{y},\;d\right)$. The ratio $R_{\text{cMLC}}$ is associated with the direct MLC transmission *T*(*d*) and MLC scatter β (*d*) parameters, and the ratio $R_{\text{dMLCgap}}$ is associated with the dosimetric gap parameter Δ*G*(*d*).

### C. Closed MLC tests

The ratio of closed MLC dose to open beam dose $R_{\text{cMLC}}$ has two contributing factors, which are related to direct MLC transmission and MLC scatter. We assume that the primary, head scatter, backscatter, and phantom scatter photons in the closed MLC measurement have properties similar to those in the open‐beam measurement, except that they are attenuated and scattered by the MLC (except the backscatter from the jaws, which does not reach the MLC). The photons transmitted and scattered by the MLC have an altered spectrum as compared with open‐beam photons, but this alteration is effectively taken into account in the depth dependence of the MLC parameters, as will be described shortly.

According to Monte Carlo simulations,^(^ ^3^ ^,^ ^4^ ^)^ MLC scatter radiation is rather uniform across the field. At an off‐axis point placed at x=7 cm, the MLC scatter contribution decreases by 5% – 3% below that on the central axis when normalized to the total transmission at the central axis for fields from 5×5 cm to 20×20 cm.^(3)^ The MLC scatter at the central axis is shown to be 3% – 23% of the total MLC transmission for fields from 5×5 cm to 20×20 cm.^(3)^ For these reasons, we adopted a uniform model of distribution of MLC scatter. We verified this approximation for off‐axis points and for asymmetric fields by performing measurements as described later in this subsection.

Thus, we adopt the following parameterization of the MLC transmission and MLC scatter:

(2(A)) $$R_{cMLC}=T(d)\left(1+\beta (d)c_{eff}^{2}\right)\;\;\text{and}$$

(2(B)) $$c_{eff}=\frac{(1+k)c_{x}c_{y}}{k\;c_{x}+c_{y}}\;\;,$$

where the MLC scatter parameter *β*(*d*) is defined as a correction to direct MLC transmission *T*(*d*), so that the total MLC transmission is ${T(d)[1+\beta (d)c_{\text{eff}}}^{2}]$, and ${c_{\text{eff}}}^{2}$ is the area of an effectively square field. This definition is similar to the definition of scatter‐to‐primary ratio used in modeling head scatter $S_{H}$ and phantom scatter $S_{P}$.^(^ ^13^ ^–^ ^18^ ^)^ Effective field size $c_{\text{eff}}$ is expressed in terms of collimator exchange factor *k*. For Varian 21 EX open‐beam data, k≈1.3. We used k=1.35 for modeling MLC transmission.

The MLC transmission is a function of depth, because, for low‐ and medium‐energy LINACs, percentage depth dose could increase by 5% – 10% because of the MLC‐induced beam hardening.^(3)^ We assume a linear depth dependence for direct MLC transmission *T*(*d*):

(3) $$T(d)=T(d_{\max})\left(1+\kappa \;(d-d_{\max})\right),$$

where κ is a beam‐hardening parameter. The specific functional form is confirmed by the experimental results. The magnitude of the MLC scatter is expected to diminish with increasing depth because of a difference in the spectrum of the photons directly transmitted by the MLC as compared with those scattered by the MLC.

### D. Dynamic MLC gap tests

We adopted a definition of fluence found in IMRT planning systems.^(^ ^5^ ^,^ ^11^ ^–^ ^12^ ^,^ ^19^ ^)^ This definition of fluence is not exactly the physical definition (the number of particles per unit area). For this reason, we use a notion of ray density ϕ, which is a mathematical concept used in IMRT optimization and dose calculation. It is essentially a density of rays in the isocentric plane emanating from a point source at a source‐to‐axis distance of 100 cm. The value of ϕ for the open beam or for static MLC is 1 inside the field and 0 outside the field (sharp edges, no penumbra).

The dynamic MLC gap dose is a combination of dose contributions from the open beam and from closed MLC. The open‐beam contribution is proportional to the direct radiation and is associated with a direct ray densityϕ in our notation. The closed MLC contribution is proportional to the radiation attenuated by the MLC and is associated with a (1 ‐ ϕ) term. In principle, direct ray density ϕ can take any value between 0 and 1 for dynamic MLC delivery.

The RFO and CMO are included in the definition of ϕ by offsetting all the leaf positions by a constant gap, ΔG/2=RFO+CMO/2. Typically, CMO is set to values close to 0. Parameter Δ*G* is called “dosimetric gap” in Eclipse.^(19)^ It effectively accounts for the non‐uniform leaf‐end transmission and remaining mechanical offset. If the design of the MLC leaf‐end is not rounded, then ΔG=CMO. For a dynamic MLC gap of size *G*, moving at a constant speed across a distance Δ*x*, the direct ray density is uniform and its value is

(4) $$\phi_{dMLCgap}=\frac{G+\Delta G}{\Delta x}.$$

Dose ratio $R_{\text{dMLCgap}}$ is the sum of the open‐beam and cMLC ray densities,

(5) $$R_{dMLCgap}=\phi_{dMLCgap}+\left(1-\phi_{dMLCgap}\right)R_{cMLC}\;\;,$$

where the first contribution is attributable to primary fluence through the MLC gaps, and the second term is the combination of transmission and scatter through the MLC.

### E. Correction of TPS fluence model

#### E.1 Model of fluence in the TPS

Consider a fluence as defined by the planning system (Eclipse) $\Phi_{\text{TPS}}$. In the current TPS fluence model, MLC transmission *T* is an effective constant for all field sizes and depths. In the present model, the total fluence is essentially a ray density $\Phi_{\text{TPS}}$,^(^ ^11^ ^,^ ^12^ ^,^ ^19^ ^)^

(6) $$\Phi_{TPS}=\phi +T_{TPS}(1-\phi )\;\;,$$

where ϕ is a general ray density. The value of ϕ for the open beam or for static MLC is 1 inside the field and 0 outside the field (sharp edges, no penumbra).

The TPS MLC parameters and the parameters used in our model are different. The TPS does not allow more than two fixed parameters ${\left(T,\Delta G\right)}_{\text{TPS}}$. The model of MLC transmission presented here has three parameters: ${\left(T,\beta ,\Delta G\right)}_{\text{exp}}$. In both models, the parameters are determined experimentally, and accordingly, they have different values. However, in a TPS, finding one set of parameters ${\left(T,\Delta G\right)}_{\text{TPS}}$ that will account for all field size, depths, and fluence complexity is difficult.

#### E.2 Correction of TPS model

Evaluating the importance of the MLC scatter for various levels of the effective ray density ‹*ϕ*› is instructive. The effective ray density ‹*ϕ*› is a spatial average of ϕ within the limits set by the jaws [ϕ=1→‹ϕ‹≡1; see also references^(^ ^11^ ^–^ ^12^ ^)^]. In the discussion that follows, we ignore the dependence on depth *d* of the MLC parameters. Assuming that the MLC scatter is spatially uniform across the field—which is a good first‐order approximation according to Kim et al.,^(3)^ Arnfield et al.,^(4)^ Chui et al.,^(5)^ and our own results—it is possible to modify the TPS model of ray density $\Phi_{\text{TPS}}$ simply by adding a MLC scatter correction term and changing the meaning of the MLC transmission parameters:

(7) $$\Phi_{new}=\phi +T_{new}(1-\phi )+T_{new}\left(1-\left\langle \phi \right\rangle \right)\beta_{new}c_{eff}^{2}.$$

This approach is clear once it is realized that a convolution of the uniform MLC scatter kernel with a ray density ϕ is equivalent to taking an average of the ray density. These relations are validated by performing central axis and off‐axis measurements for sweeping gap and for complex IMRT plans.

#### E.3 A scaling law

In clinical practice, approximate scaling laws are used for various dosimetric estimations. For this reason, we provide a crude estimate of MLC scatter in an IMRT field in terms of ‹*ϕ*›. Based on equation 7, the relative contribution of the MLC scatter for an IMRT field is

(8) $$\frac{\left\langle \Phi_{new}\right\rangle -{\left\langle \Phi_{new}\right\rangle }_{\beta =0}}{\left\langle \Phi_{new}\right\rangle }=\frac{T_{new}\beta_{new}c_{eff}^{2}}{\left\langle \Phi \right\rangle /\left\langle 1-\phi \right\rangle +T_{new}\left(1+\beta_{new}c_{eff}^{2}\right)}.$$

The advantage of this calculation is that it shows how the MLC scatter contribution depends on the complexity of the IMRT plan, expressed by spatially averaged ray density ‹*ϕ*›—a “scaling law” in terms of ‹*ϕ*›.

### F. The OB–cMLC–dMLCgap tests

#### F.1 MLC parameters

Direct MLC transmission *T*(*d*) and MLC scatter *β*(*d*) parameters are determined from equations 2(A) and 2(B) and the measured dose ratio $R_{\text{cMLC}}$ for various field sizes. The dosimetric gap Δ*G*(*d*) is determined independently from the other MLC parameters using equations 4 and 5 and the measured dose ratios $R_{\text{cMLC}}$ and $R_{\text{dMLCgap}}$. We used the Levenberg–Marquardt fitting method in Mathematica (Wolfram Research, Champaign, IL). Experimental methods used in determination of MLC parameters and application of the model of MLC transmission to dynamic IMRT are described next.

#### F.2 Determination of MLC parameters with OB–cMLC–dMLC tests

**Figure 1:**
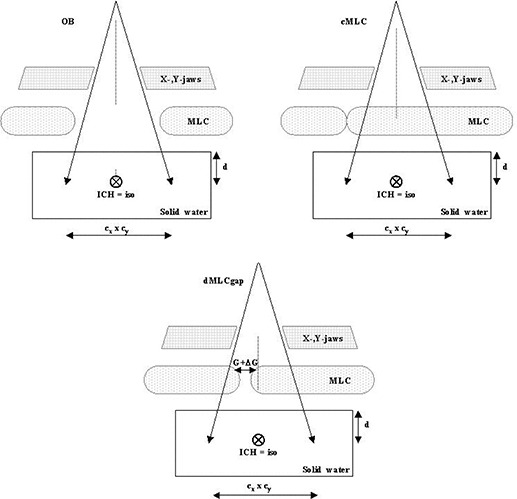
Schematic of the open beam (OB)–closed multileaf collimator (cMLC)–sweeping gap (dMLCgap) tests. The depth *d* and field size $c_{x}\times c_{y}$ are the same for all three cases. The detector (ICH) is placed at the isocenter (iso), perpendicular to the beam. From the above measurements, $R_{\text{cMLC}}$ and $R_{\text{dMLCgap}}$ dose ratios are determined.

The OB, cMLC, and dMLCgap tests were performed for a selected combination of rectangular (jaw) field sizes $c_{x}\times c_{y}$, at a given depth *d*, in a Solid Water phantom at the machine isocenter, and for a given beam energy (Fig. 1). The dMLCgap test was performed for various constant gap sizes *G*, traveling at a constant speed across a distance $\Delta_{x}=c_{x}+G$.

Specifically, the following irradiation conditions were applied on Varian 6EX (6 MV) and 21 EX (6 and 10 MV) LINACs equipped with Millennium MLCs:

- Field sizes $c_{x}\times c_{y}=5\times 5\;\text{cm}$, 7×7 cm, 10×10 cm, 12×12 cm, 12×20 cm, 12×28 cm
- dMLC gaps *G* of 0.1 cm, 0.5 cm, 1.0 cm, 2.0 cm
- Depths of $d=d_{\text{max}}$ of 5 cm, 10 cm, where $d_{\text{max}}$ is 1.5 cm for 6 MV and 2.5 cm for 10 MV

The measurements were performed at the central axis with an ionization chamber—Exradin A12 (Standard Imaging, Middleton, WI) having a 0.6 cm^3^ active volume—placed at isocenter perpendicular to the beam and perpendicular to the MLC leaves. The detector size was sufficiently large to average the interleaf and intraleaf transmissions during measurement. Thus, the interleaf leakage effect for the cMLC and dMLCgap tests is effectively incorporated into the MLC parameters. The tongue‐and‐groove effect is not present in the cMLC or dMLCgap tests (the tongue or groove leaf sides are not exposed to radiation separately).

#### F.3 Filtering of the scatter with stereotactic cones

Additional open beam and closed MLC measurements were performed to separate the MLC scatter signal from the MLC transmission signal in the $R_{\text{cMLC}}$. These measurements were performed by placing an ionization chamber with a buildup cap in air and irradiating the detector twice—with and without a stack of collimating cones (stereotactic cones). Lead cones (of diameters 2.75 – 3.25 cm) in a 38‐cm stack were placed immediately above the detector. An ionization chamber TDC100 with a water‐equivalent buildup cap of 1 cm thickness was used. Otherwise the setup was the same as that shown in Fig. 1.

#### F.4 MLC transmission at off‐center points, inside and outside of field for symmetric and asymmetric fields

The OB‐cMLC‐dMLCgap tests as a function of field size and depth were measured for symmetric field sizes $\left(x1=x2=c_{x}/2,y1=y2=c_{y}/2\right)$ at the field center. To test the variation of MLC scatter at off‐center points inside and outside of field for symmetric and asymmetric fields, additional open beam and closed MLC tests were performed for various asymmetric jaw settings. Jaw sizes were chosen to depict extreme jaw positions within the limits imposed by dynamic MLC delivery (Table 1).

**Table 1:** Jaw settings for tests of multileaf collimator scatter at off‐center points inside and outside of field for symmetric and asymmetric fields

		Jaw settings (cm)	
*x*1	*x*2	*y*1	*y*2
*Symmetric fields*			
2.5	2.5	2.5	2.5
5	5	5	5
7	7	7	7
7	7	10	10
7	7	15	15
7	7	19	19
*x‐jaws symmetric, y‐jaws asymmetric*			
7	7	15	19
7	7	11	19
7	7	7	19
7	7	3	19
*y1‐jaw covers the detector, x‐jaws symmetric*			
7	7	−3	19
7	7	−7	19
7	7	−10	19
7	7	−10	14
7	7	−10	10
*x2‐jaw covers the detector, y‐jaws symmetric*			
16	−2	2.5	2.5
16	−2	5	5
16	−2	7	7
16	−2	9	10
16	−2	15	15
16	−2	19	19
*x2‐jaw covers the detector, y‐jaws asymmetric*			
16	−2	15	19
16	−2	11	19
16	−2	7	19
16	−2	3	19
*x2‐jaw and y1‐jaw cover the detector*			
16	−2	−3	19
16	−2	−7	19
16	−2	−10	19
16	−2	−10	14

These measurements were taken both inside and outside of the field in air with the A12 ionization chamber inside an aluminum buildup cap of d=1.5 cm. Contributions from jaw transmission outside of the field were subtracted from the total ionization chamber signal as described next. Otherwise the setup was similar to that shown in Fig. 1.

We used the dose ratios that follow as figures of merit to compare MLC scatter calculated according to the model described earlier [uniform MLC transmission in equations 2(A) and 2(B)] with the ionization chamber measurements. For points measured inside the field, a dose ratio of $R^{\text{in}}{}_{\text{cMLC}}$ is sufficient to describe MLC transmission:

(9(A)) $$R_{cMLC}^{in}=R_{cMLC}^{off}.$$

However, for points outside of field, no direct MLC transmission but extra jaw transmission occurs. The jaw transmission adds an offset signal and is subtracted from the total dose ratio $R^{\text{off}}{}_{\text{cMLC}}$ to obtain $R^{\text{out}}{}_{\text{cMLC}}$, which properly characterizes MLC transmission outside the field:

(9(B)) $$R_{cMLC}^{out}=R_{cMLC}^{off}-R_{cMLC}^{off}\left(c_{\text{eff}}\to 0\right)\;\;,$$

where the total (auxiliary) dose ratio was determined from the measurements

(9(C)) $$R_{cMLC}^{off}=\frac{D_{cMLC}\;(x,y,x_{1},x_{2},y_{1},y_{2})}{D_{OB}\;(0,0,-c_{x}/2,c_{x}/2,c_{y}/2)},$$

and term $R_{cMLC}^{off}\left(c_{eff}\to 0\right)$ is an extrapolation to zero field size for asymmetric jaws. The above dose ratios $R^{\text{in}}{}_{\text{cMLC}}$ and $R^{\text{out}}{}_{\text{cMLC}}$ are exactly the same as $R_{\text{cMLC}}$ (defined by equation 2) for symmetric fields at the central axis, but they are expected to be smaller than $R_{\text{cMLC}}$ for asymmetric fields (or for off‐center points) because of a gradual fall‐off of the MLC scatter with the increasing off‐center distance.

#### F.5 Dynamic MLC gap tests outside of field

Strictly speaking, our model of MLC transmission applies to IMRT at the central axis. Field‐size dependence is shift invariant in our model, which is an approximation. To test the validity of our beam model outside of field, additional open beam and dynamic MLC gap tests were performed using an ionization chamber for a limited number of cases. Specifically, the ionization chamber was placed at x=0 cm, 7 cm, and 10 cm. The field sizes $c_{x}\times c_{y}$ were 10×10 cm and 14×30 cm. Depth *d* was 10 cm, and energy was 6 MV. Otherwise, the setup was similar to that shown in Fig. 1.

#### F.6 Magnitude of MLC scatter for complex IMRT: central axis

To measure the magnitude and uniformity of MLC scatter for complex IMRT plans, we performed measurements in solid water with an A18 ionization chamber (0.125 cm^3^) at a depth of 1.5 cm at the central axis. The IMRT plan consisted of 23 fluences (9 fields requiring 23 split fluences), with an MU efficiency of about 8%. The field sizes were large $\left(c_{x}\approx 14\;\text{cm},c_{y}\approx 34\;\text{cm}\right)$, and the treated volume was large (≈30×30×34 cm). Thus, the MLC transmission and scatter contributions were sufficiently significant to test the spatial dependence for complex IMRT.

We performed two series of measurements with various magnitudes of total scatter component: one for large *y*‐jaw settings $\left[y1=y2=17\;\text{cm}(c_{y}=34\;\text{cm})\right]$ and another for cropped *y*‐jaws $\left[y1=y2=2.5\;\text{cm}(c_{y}=5\;\text{cm})\right]$. Doses were calculated with a uniform MLC scatter component (β> 0) and without MLC scatter (β=0) according to equation 7.

We compared measured with calculated relative IMRT doses: ${\left[(D_{34\text{cm}}-D_{5\text{cm}})/D_{34\text{cm}}\right]}_{\text{exp}}$ compared with ${\left[(D_{34\text{cm}}-D_{5\text{cm}})/D_{34\text{cm}}\right]}_{\beta =0}$ and $[(D_{34\text{cm}}-D_{5\text{cm}})/{D_{34\text{cm}}]}_{\beta =\beta }$. The values of the MLC parameters (T,β,ΔG) were determined from the OB–cMLC–dMLCgap test and the MLC scatter dose was calculated according to equation 2. By using the respective dose ratios $\left[(D_{34\text{cm}}-D_{5\text{cm}})/D_{34\text{cm}}\right]$, we minimized the effects attributable to dose gradients. Otherwise the experiment was the same as that shown in Fig. 1.

#### F.7 Magnitude of MLC scatter for complex IMRT: two‐dimensional

In addition, we performed measurements with an ionization chamber array (MatriXX: Wellhofer, Bartlett, TN) for a clinically relevant case. Measurements were performed and analyzed at d=15 cm for a gantry angle of 0 degrees. An IMRT plan composed of 9 fields was split into 23 subfields. The sizes of the original fields were large, covering an area of about 30×30 cm. We compared measured doses to TPS doses and to our calculations of MLC scatter using equations 7 and 8. We limited analysis to an area of 24×24 cm (the size of the detector) and determined total dose per field and total dose for the composite plan within the 24×24 cm area. Total (or average) dose per field and per plan is a good descriptor of the magnitude of the MLC scatter for the reasons outlined in the next paragraph.

Comparison of two‐dimensional (2D) doses with output from a TPS for clinically relevant cases is possible. However, interpretation requires caution. If a large fluence map is split into 2 or 3 subfields, the dose bias is additive in the composite fluence as well as in the composite plan. Because the MLC scatter is more or less uniform, but IMRT fluences for complex plans are highly irregular and show many low‐dose regions, analysis of dose differences attributable to MLC scatter are preferably stated in terms of total dose within the field (or average dose). This dose bias is seen more clearly in a composite plan, which is more uniform.

### G. IMRT planning system

#### G.1 Calculation of doses using the TPS

Experimental OB–cMLC–dMLCgap tests were simulated using an IMRT TPS (Eclipse version 7.3.1), and doses were calculated for the same setups. Dose points and profiles were exported so that they could be compared with the measured data. The plans were calculated for the dMLC parameters currently used clinically. For instance, for the energies considered, the TPS parameters used in our department are $T_{\text{TPS}}=1.4\%,1.4\%$, and 1.7%, and $\Delta G_{\text{TPS}}=0.175$, 0.175, and 0.190. Parameter $\beta_{\text{TPS}}$ is not defined in the TPS. A dose rate *R* of 400 MU per minute was used. The ray density calculation in the TPS depends on the dose rate, because the MLC speed is finite, which must be taken into account.

In the OB–cMLC–dMLCgap test, we consider more parameters (T,β,ΔG), which depend on depth. These parameters must be considered together. As a result, a comparison of the numeric value of the TPS MLC parameters to the numeric values of the parameters in our model makes little sense. The doses resulting from the application of the two models are better quantities for such a comparison.

### H. Error analysis

To see the relationship between the uncertainties in the MLC parameters and the relative fluence errors, we consider a general case. In the discussion that follows, δ refers to the uncertainty in the determination of the MLC parameters (T,β,ΔG) and ray density Φ.

Generally, the relative ray density error attributable to the uncertainty δ*T* in the determination of the MLC transmission *T* can be evaluated using a formula from Zygmanski et al.^(11)^

(10(A)) $${\left(\frac{\left\langle \delta \Phi \right\rangle }{\left\langle \Phi \right\rangle }\right)}_{T}≅\delta T\frac{1-\frac{\left\langle G\right\rangle }{\Delta x}}{\frac{\left\langle G\right\rangle }{\Delta x}}≅\delta T\frac{\Delta x}{\left\langle G\right\rangle },$$

and the errors attributable to the uncertainty in the dosimetric gap δ(Δ*G*) [that is, δ(ΔG)=(2ΔRFO+CMO)] from a formula by Zygmanski et al.,^(12)^

(10(B)) $${\left(\frac{\left\langle \delta \Phi \right\rangle }{\left\langle \Phi \right\rangle }\right)}_{\Delta G}≅\frac{\delta (\Delta G)}{\left\langle G\right\rangle }.$$

From the foregoing, if <G> is of the order 0.5 – 1.0 cm, and Δ*x* is 10 – 15 cm, even small uncertainties—a δ*T* of 0.001 or a δ(Δ*G*) of 0.05 cm—contribute significantly to the ray density errors. These contributions have even greater importance in the regions outside the target and within the organs at risk, where the fluence is generally relatively low.

## III. RESULTS

### A. Closed MLC tests

**Figure 2:**
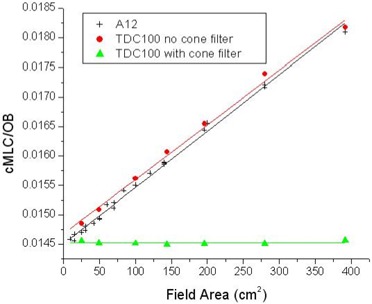
An example of the measured (data points) and fitted (straight lines) dose ratios for closed multileaf collimator $\left(R_{\text{cMLC}}\right)$ with and without a cone for the 6‐MV 6EX linear accelerator. Measurements were made in air with the cone (filled triangle), in air without the cone (filled cross), and in solid water at depth $d_{\text{max}}$ without the cone (solid circle). Ionization chambers A12 and TDC100 were used. The discrepancy between the two sets of data without the cone (solid cross and solid circle) is within the accuracy of the method.

Fig. 2 shows an example of $R_{\text{cMLC}}$ (ratio of closed MLC dose to open‐beam dose) as a function of the field area ($c_{x}\times c_{y}$). Measurements were made in air with and without cones to show that MLC scatter is responsible for the field‐size dependence of $R_{\text{cMLC}}$. By placing a stack of cones above the detector, the MLC scatter was effectively removed.

In addition, Fig. 2 shows a comparison of $R_{\text{cMLC}}$ measured without cones in Solid Water at $d_{\text{max}}$ and $R_{\text{cMLC}}$ measured in air without cones. These two data sets are the same to within the uncertainty of the measurements, indicating that phantom scatter does not bias the derivation of the MLC parameters because it is effectively canceled when the dose ratio is taken. Based on the in‐air measurements with and without cones, head scatter in open beam is about 4%, and the field‐size dependence of the closed MLC is about 25% for the field sizes being considered (5 – 20 cm). The closed MLC dose includes a head scatter contribution similar to the open‐beam dose. However, only about 21% of the field size dependence of MLC transmission is attributable to scatter from the MLC. That finding confirms that the MLC scatter parameter β does not include head scatter.

Table 2 shows the MLC parameters derived from the OB–cMLC–dMLCgap test for $d=d_{\text{max}}$, 5 cm, 10 cm, and two Varian LINACs (6EX and 21EXs). The beam‐hardening parameters κ, based on equation 3, are $0.0075\;{\text{cm}}^{-1}(6\;\text{MV}\;6\text{EX}),0.010\;{\text{cm}}^{-1}(6\;\text{MV}\;21\text{EXs})$, and $0.0027\;{\text{cm}}^{-1}$ (10 MV 21EXs). Because the TPS does not model the MLC scatter explicitly, direct comparison of values from the OB–cMLC–dMLCgap test (*T*, β, Δ*G*) to the TPS values may be misleading.

**Table 2:** Parameters determined from tests of open beam, closed multileaf collimator, and dynamic MLC gap as a function of depth (d) for various machines (6EX and 21EXs linear accelerators: Varian Medical Systems, Palo Alto, CA) and energies (6 MV, 10 MV)

*d* (cm)	$T_{\text{exp}}$			${\left(\beta \right)}_{\text{exp}}(10^{-4}{\text{cm}}^{-2})$			$\Delta G_{\text{exp}}$ (cm)		
	6EX (6 MV)	21EXs (6 MV)	21EXs (10 MV)	6EX (6 MV)	21EXs (6 MV)	21EXs (10 MV)	6EX (6 MV)	21EXs (6 MV)	21EXs (10 MV)
$d_{\text{max}}$	0.0144	0.0139	0.0170	6.4	6.5	6.2	0.146	0.157	0.174
5 cm	0.0147	0.0144	0.0171	5.8	5.8	6.2	0.147	0.159	0.176
10 cm	0.0153	0.0151	0.0174	5.1	5.0	5.7	0.148	0.162	0.177
δ(T,β,ΔG)	δT=±0.00025	$\delta \beta =\pm 0.2\times 10^{-4}{\text{cm}}^{-2}$	δ(ΔG)=±0.005 cm

T=direct multileaf collimator transmission; β=MLC scatter correction parameter; ΔG=dosimetric gap(2×RFO+CMO)].

The numeric results are consistent with Monte Carlo studies for a similar LINAC,^(3)^ except that the Monte Carlo data for 18 MV indicate that MLC scatter relative to MLC transmission is lower than it is for 6 MV. Apparently, 10 MV is not sufficiently different as compared with 6 MV to significantly decrease the MLC scatter contribution.

### B. Dynamic MLC gap tests

**Figure 3:**
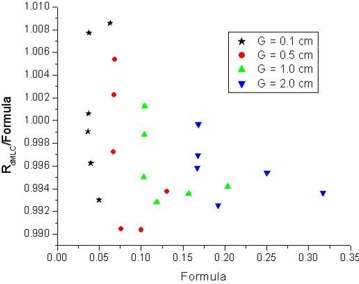
Example of the measured sweeping gap dose ratio $\left(R_{\text{dMLCgap}}\right)$ divided by the right side of equation 5 (“formula”) for four gap sizes *G* and six field sizes $\left(c_{x},c_{y}\right)$ for depth d=10 cm. “Formula” is equal to $\left[\phi_{\text{dMLCgap}}+(1-\phi_{\text{dMLCgap}})R_{\text{cMLC}}\right]$ and is derived from measurements and from equation 4. Derivation of the dosimetric gap Δ*G* is based on equation 5. As Table 2 shows, the dosimetric gap is practically constant for various field sizes and depths. OB =open beam; cMLC = closed multileaf collimator.

The dosimetric gap Δ*G* is determined from equations 4 and 5 independently from the MLC transmission and scatter parameters. The results shown in Fig. 3 validate the methodology.

Fig. 3 shows an example of the measured dose ratio $R_{\text{dMLCg}}$ divided by the right side of equation 5, derived from measured OB and cMLC data for various gap sizes (*G*) and field sizes $\left(c_{x},c_{y}\right)$, for d=10 cm. The data are contained within a ±1% window. This disagreement is attributable in part to noise and in part to other inherent uncertainties described in the next subsection. However, that disagreement has a negligible effect on the model parameters and potential dose errors that may result. (See the error discussion subsection.)

### C. MLC parameters

Table 2 shows the MLC parameters determined from the OB–cMLC–dMLCgap tests, together with uncertainties in their determination. The uncertainties include noise in the input data to the fitting programs, and other systematic uncertainties attributable to the MLC file itself, the choice of detector, and reproducibility. These uncertainties result in uncertainties of ray density according to equations 10(A) and 10(B):

- Δx/ G = 10−20 and δT=±0.00025→ΔΦ/Φ=±(0.25% to 0.5%);
- G=0.5 cm−1.0cm and δΔG=±0.005 cm→ΔΦ/Φ=±(0.5% to 1.0%);
- Δx/ G=10−20,c=20 cm and $\delta \beta =\pm 0.2\times 10^{-4}{\text{cm}}^{-2}\to \Delta \Phi /\Phi =\pm (0.2\%\;\text{to}\;0.3\%)$.

The uncertainty in *T* and β add in the formula 2(A). Misalignment of the MLC (CMO) is automatically included in Δ*G* and is not considered to be a part of the inherent uncertainty of the method.

### D. Non‐uniformity of MLC scatter

**Figure 4:**
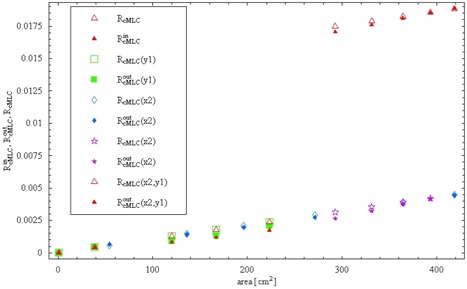
Measured compared with calculated total multileaf collimator (MLC) transmission (direct and scatter) for various *x* and *y* jaw positions as a function of field area. Dose ratios (*R*) were measured for the Varian 6EX (6 MV) in air with an A12 ionization chamber and aluminum buildup cap. For the points outside the field, (*x*2), (*y*1), and (*x*2,*y*1) indicate the jaw or jaws behind which the ionization chamber was placed.

Fig. 4 shows the field size dependence of calculated $R_{\text{cMLC}}$ according to equations 9(A), 9(B), and 9(C), and measured $R^{\text{in}}{}_{\text{cMLC}},R^{\text{out}}{}_{\text{cMLC}}$ dose ratios. As expected, $R_{\text{cMLC}}$ is a little bit larger than $R^{\text{in}}{}_{\text{cMLC}}$ and $R^{\text{out}}{}_{\text{cMLC}}$ for all combinations of jaw sizes. However, the field size dependence is stronger than the dependence on the distance to field center, indicating that a model of uniform MLC scatter gives a good estimate (an upper limit) of MLC scatter.

### E. Dose profiles for dynamic MLC gap patterns

**Figure 5:**
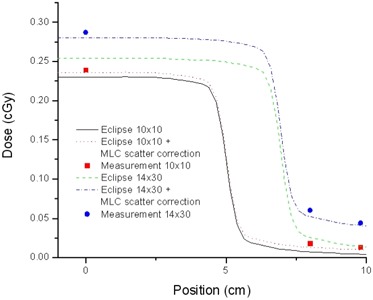
Absolute dose profiles for the treatment planning system and ionization chamber measurements (points) for an extreme sweeping gap of 0.6 mm width, for two field sizes: 10×10 cm and 14×30 cm. Also shown are profiles corrected using equations 4, 5, and 7.

Fig. 5 compares the dynamic MLC gap test with measurement for the TPS dose profiles and the profiles corrected to account for MLC scatter (three points). Correction attributable to MLC scatter was derived from equations 4 and 5 and was applied the same way at the central axis and at off‐axis points inside and outside the field according to equation 7. The corrections in the absolute doses outside the field are comparable to the differences at the central axis because MLC scatter is relatively uniform; points outside the field receive almost the same amount of MLC scattered radiation. As expected, based on equations 4 and 5, the MLC scatter effect is stronger for smaller gaps and larger field sizes. Experimental validation of the transmission model for arbitrary IMRT fluence patterns is described next.

### F. Magnitude of the MLC scatter for clinical cases

#### F.1 Dose difference (cy=34 cm as compared with 5 cm) with and without MLC scatter

**Figure 6:**
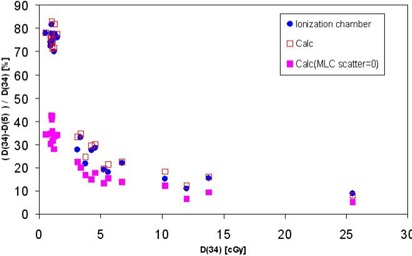
The calculated dose $\left[(D_{34\text{cm}}-D_{5\text{cm}}/D_{34\text{cm}}\right]$, with and without multileaf collimator (MLC) scatter, compared with the absolute dose $D_{34\text{cm}}$ per field determined by measurement. The $D_{34\text{cm}}$ signifies dose per field for an intensity‐modulated radiation therapy plan with $c_{y}=34\;\text{cm}$; the $D_{5\text{cm}}$ signifies dose per field for the same plan with *y* jaw cropped to 5 cm.

Fig. 6 shows experimental and calculated dose differences: ${\left[(D_{34\text{cm}}-D_{5\text{cm}})/D_{34\text{cm}}\right]}_{\text{exp}}$ as compared with ${\left[(D_{34\text{cm}}-D_{5\text{cm}})/D_{34\text{cm}}\right]}_{\beta =0}$ and ${\left[(D_{34\text{cm}}-D_{5\text{cm}})/D_{34\text{cm}}\right)}_{\beta =\beta }$. The total measured doses for a cumulative plan were about 110 cGy $\left(c_{y}=34\;\text{cm}\right)$ and 85 cGy $\left(c_{y}=5\;\text{cm}\right)$. The calculated doses attributable to direct MLC transmission were about 25% (34 cm) and 30% (5 cm), and because of MLC scatter, 12% (34 cm) and 2% (5 cm). The MLC scatter can be seen to be especially large for low‐dose fields (ionization chamber outside of a specific subfield). In fact, MLC scatter is about inversely proportional to the total dose, and it is more or less equal to the total head scatter dose plus phantom scatter dose from regions outside of $c_{y}=5\;\text{cm}$ at d=1.5 cm.

#### F.2 2D verifications

We compared the measured MatriXX doses to TPS doses and to our calculations of MLC scatter. Measured fluence maps were found to be very similar in shape to the TPS maps, but a small dose bias was observed. Total dose bias per field ranged from +6.4% to +9.1%, and was +7.5% for the composite plan dose. Our calculation of the magnitude of MLC scatter according to equations 7 and 8 resulted in very similar numbers (+4.2% to +10.5% per field, and +7.8% for composite plan). This finding shows that the discrepancy between the TPS doses and measurement comes predominantly from MLC scatter. The small discrepancies between our estimation of MLC scatter and the experimentally determined dose biases for individual fields may be the result of dose gradients (averaging effect of finite‐size detectors) and the presence of tongue‐and‐groove effects.

#### F.3 Scaling law

In routine IMRT QA for less complex cases, the sorts of effects discussed in the preceding subsection are difficult to demonstrate in a single fluence with an ionization chamber (insufficiency of data points and the problem of gradient), or with a 2D detector (problem of film normalization). The cumulative measurement—including all the fields and using an ionization chamber—should reveal this sort of discrepancy, but not necessarily its nature (MLC scatter origin).

For this reason, we present an approximate scaling law formula (equation 8) that describes how to calculate effective fluence error as a function of average fluence level. This formula may be helpful in predicting the MLC scatter effect on IMRT plans of various complexity. Plan complexity, dose output efficiency, and the effective MLC gap size are all related.^(^ ^11^ ^,^ ^12^ ^)^

**Figure 7:**
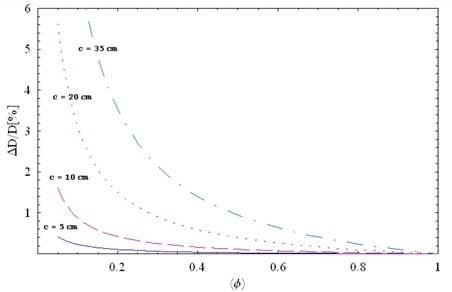
A relative magnitude of multileaf collimator scatter (based on equation 8) as a function of the effective ray density ‹*ϕ*› for square jaw sizes: 5 cm (solid line), 10 cm (long dashed line), 20 cm (dotted line). The *T* was set to 0.015, and β, to 0.0006. ΔD=change in absolute dose; D=absolute dose.

Fig. 7 shows the relative magnitude of MLC scatter as a function of ‹*ϕ*›. For small field sizes (c=5 cm or 10 cm), the magnitude of MLC scatter is small, but for larger sizes (c=20 cm), MLC scatter is small only when ‹*ϕ*› exceeds 0.3. When ‹*ϕ*› equals 0.3, the implication is that about 3 times the MUs (1 / 0.3) are used for IMRT delivery as for an open beam with the same depth and field size. In practice, ‹*ϕ*› can be as small as 0.1 for complex IMRT plans. If the field size is large for these cases, the magnitude is significant.

## IV. DISCUSSION

### A. General

In the commissioning of an IMRT planning system, the OB–cMLC–dMLCgap tests can be performed at one clinically relevant depth for a smaller number of field sizes (for example, 5×5 cm, 10×10 cm, 12×28 cm) and MLC gaps (for example, 0.1 cm, 1.0 cm) than were used in this study. This amounts to about 3×3×2(=18) measurements.

Our study is complementary to previous reports.^(^ ^3^ ^,^ ^4^ ^,^ ^10^ ^)^ In our method, in addition to the dMLCgap and OB tests, we performed cMLC tests and described MLC properties in terms of three MLC parameters (different figures of merit) derived from the measured data: ratios of cMLC dose to OB dose and ratios of dMLCgap dose to OB dose. Based on published Monte Carlo simulations for high‐beam energy (15 MV),^(3)^ the MLC scatter relative to MLC transmission decreases considerably. However, as our study shows, the scatter at 10 MV is almost the same as at 6 MV.

More studies are needed to understand the effect on real clinical cases of the field‐size and depth dependence of total MLC transmission—especially the effect on normal organs, which are often in the low‐fluence areas and for which the relative dose error might therefore be larger than for the target. The confounding factors are the non‐uniformity of the fluences and the fact that they are split unevenly (into 2 or 3 sections, with overlap in various places), the patient's anatomy (relative position of the target with respect to normal organs), and the location of inhomogeneities. Equation 7 is still valid for clinical cases to the degree that the MLC scatter is uniform and can be represented by ‹*ϕ*›. A more accurate description would involve using (*x, y*)‐dependent MLC scatter in a manner similar to that used in previously reported work.^(^ ^13^ ^–^ ^18^ ^)^

Small out‐of‐field dose errors for the open beams or static MLC conformal plans attributable to inadequate penumbra modeling (different from the MLC aperture effects discussed in this report) may not be clinically significant. However, in complex IMRT with split fluences, these small errors may be magnified or may add up to significant dose errors, especially to the normal organs, as our study shows. For these reasons, we recommend that, for complex IMRT plans, low ‹*ϕ*› doses outside of the isocenter and outside of the target be measured (for instance, with several films placed in various slices in a phantom).

We suggest that an ideal IMRT planning system should include MLC scatter parameters and should permit the user to easily commission the system in the beam configuration using the concepts described above for cMLC and dMLCgap (or similar). Conceivably, the standard beam data [percentage depth dose PDD, tissue‐phantom ratio $S_{p}$, head‐scatter ratio $S_{H}$, off‐axis ratio OAR, and so on], which form the core of the TPS, could be extended to include the MLC scatter factor $S_{\text{MLC}}$ in a manner similar to collimator backscatter $\left(S_{\text{BS}}\right)$ and head scatter $S_{H}$. In severely complex IMRT, it may be important to include the depth dependence of the transmission and the depth dependence of the MLC scatter—although implementing those dependencies in the dose calculation algorithm in the presence of inhomogeneities would be more difficult.

We have not performed measurements for other MLC designs. However, we expect that our model, with minor modifications, would nevertheless apply to other MLC designs and LINACs. The model parameters would change in value. For instance, for MLC without rounded leaf‐ends, ΔG=0. The situation is similar to the modeling of head scatter. Further, if the MLC scatter becomes more pronounced or more dependent on off‐axis distance (or both) than it is for the Varian MLC, there could be a need to introduce a Gaussian model of MLC scatter distribution, resulting in a ${\beta (d)\;\text{Erf}(c/\Lambda )}^{2}$ correction^(16)^ instead of $\beta (d)c^{2}$ as used in our model. However, whether these modifications are necessary would have to be determined experimentally.

### B. Choice of optimal TPS parameters and potential dose errors

The TPS has only two parameters ${\left(T,\Delta G\right)}_{\text{TPS}}$, but more parameters ${\left(T,\beta ,\Delta G\right)}_{\text{exp}}$ are needed to more fully characterize dosimetric MLC properties, which depend on field size and depth. It is not possible to find one set of parameters ${\left(T,\Delta G\right)}_{\text{TPS}}$ that would work for all field sizes and all depths. Thus, a set of parameters ${\left(T,\Delta G\right)}_{\text{TPS}}$ must be found that minimizes the dose errors attributable to MLC scatter. One option is to create multiple machines with various values of ${\left(T,\Delta G\right)}_{\text{TPS}}$ in the TPS: one for small‐field IMRT $\left(c_{x}\times c_{y}\leq 10\times 10\;\text{cm}\right)$, and another for large‐field IMRT $\left(c_{x}\times c_{y}\geq 14\times 20\;\text{cm}\right)$. That approach may not be practical, however.

**Figure 8:**
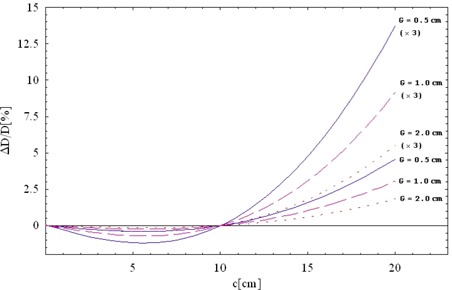
Magnitude of dose errors attributable to multileaf collimator (MLC) scatter as a function of square field size *c* when the effective treatment planning system (TPS) parameters ${\left(T,\text{xxD}G\right)}_{\text{TPS}}$ are determined for 10×10 cm (according to equation 6) and dose is calculated by the TPS for other field sizes *c* without the additional MLC scatter correction. Gap sizes (*G*) are 0.5 cm (solid line), 1.0 cm (dashed line), 2.0 cm (dotted line). Sweeping gaps travel distances of (c+G). (x3) denotes the total error when a larger field is split into three smaller fields. ΔD= change in absolute dose; D=absolute dose; G=dosimetric gap; c = the size of a split subfield.

Fig. 8 shows the approximate dose errors for a situation in which the effective TPS parameters ${\left(T,\Delta G\right)}_{\text{TPS}}$ are determined for a 10×10−cm field (according to equation 6) and are used in the TPS for dose calculation for other field sizes, neglecting the MLC scatter effect. Gap sizes *G* are 0.5 cm, 1.0 cm, and 2.0 cm, and the dose error is considered in the high‐dose region. In such a case, the dose discrepancy for the field sizes above about 15×15 cm is quite large. Interpretation of Fig. 8 requires caution.

If the field‐size dependence of MLC transmission is neglected, the large field size and the low MU efficiency are what lead to significant dose error. Each split field contributes more or less the same amount of the undesired MLC scatter dose to the in‐field and out‐of‐field regions. Because the split fields are smaller in size than the original fields (for example, three 14×35−cm fields instead of one 35×35 cm field), an impression may be created that the MLC scatter effect is less significant in the total plan. That impression is not true, because the significant factor is the field size of the original field before splitting, which tends to be large for large targets.

For these reasons, Fig. 8 shows three additional curves. The curves denoted by (*x*3) refer to an extreme case in which a large field is split into three subfields. Because the errors from the individual subfields add up, the total error is as much as three times larger. Because the actual MLC scatter decreases slightly with the off‐axis distance (Fig. 4), errors in Fig. 8 show the upper limit for dose errors attributable to MLC scatter alone.

Another crucial point is that, for split fields, the extra dose affects both the target and the normal‐organ regions, but the normal organs are more sensitive than the target to the same amount of extra dose. Fig. 8 shows relative dose error in the high‐dose region (target region). Relative dose errors in low‐dose regions are considerably larger. Because complex IMRT optimization is pushed to the limits, and because normal organs often receive the maximum allowed dose, that dose bias becomes even more significant.

### C. Other MLC effects

Analysis of the OB–cMLC–dMLCgap test presented here assumes that the detector is sufficiently large that the interleaf and intraleaf transmissions are averaged during measurement. Because all the leaves of a sweeping gap are moving in the same fashion, there is no tongue‐and‐groove effect in the OB–cMLC–dMLCgap tests. The tongue‐and‐groove effect would be present if the leaf sides of individual leaves were exposed to radiation. However, for delivery of IMRT to the patient, (i) the interleaf versus intraleaf transmission and (ii) the tongue‐and‐groove effect are enhanced when MU efficiency is low, and the leaf sides are often exposed during the leaf motion. Both of these effects result in an undesired modification of the ideal fluence. Although (i) is effectively included in the notion of the spatially averaged MLC transmission *T* (equation 2), (ii) may cause an overall underdosage of the target that can be numerically estimated only if the tongue, or groove, or both, of the MLC transmissions are directly included in the fluence calculations. This negative dose bias may be significant when fluence delivery is complex.^(20)^ Furthermore, even though use of non‐coplanar IMRT fields or variation in the collimator angle can blur the effects of both (i) and (ii), it cannot alter the total dose deposited within the target because of (ii). This aspect of complex IMRT delivery will be the focus of a future study.

We have assumed that MLC backscatter into the monitor chamber is negligible. The reason is that the backscatter from the upper jaw is not more than 2% for Varian LINACs,^(6)^ and therefore it should be significantly less for the MLC. In fact, backscatter is below the uncertainty of our experiment (1%). Should MLC backscatter play a more significant role, we would have to include a new correction factor parameter $\left(-\alpha_{\text{MLC},\text{BS}}\right)$ in equation 3.

## V. CONCLUSIONS

Ignoring the field‐size dependence of MLC transmission may lead to underestimation of dose inside and outside the field in the treatment of large targets. The exact amount of MLC scatter dose depends on the total field size before splitting and on MU efficiency, which can be described in terms of effective ray density ‹*ϕ*›. For dMLCgap tests used in the report, ‹ϕ›=(G+ΔG)/(c+G). The undesired dose bias attributable to MLC scatter from split fields (inside and outside the split field) add up in the total plan. For clinically relevant IMRT plans, MLC scatter is found to reach up to about 10% of the total dose within the target and more outside of the target. The latter increase in dose may be more important—for instance, in treatment of thorax, lung sensitivity to low doses is important when large lung volumes are being irradiated.

We have presented a phenomenologic model of beam transmittal through a MLC, and we have provided MLC tests (OB–cMLC–dMLCgap) that can be used to determine the model parameters. The model can then be used in the commissioning of a TPS or in the development of new dose‐calculation algorithms that would directly model MLC scatter and beam hardening. If the dose calculation algorithm is modified according to our model, the inclusion of the additional parameters would improve the accuracy of dose estimation.

## ACKNOWLEDGMENTS

We express our gratitude to Mike Makrigiorgos for offering dosimetry support.

## Supporting information

Supplementary Material Files

Supplementary Material Files
