# Supplementary material for: Determination of depth and field size dependence of multileaf collimator transmission in intensity‐modulated radiation therapy beams
Source: J Appl Clin Med Phys. 2007 Oct 24;8(4):76–95. doi: 10.1120/jacmp.v8i4.2693 (PMC5722617; doi:10.1120/jacmp.v8i4.2693)
Supplement: Supplementary file 2 — Supplementary Material Files [file ACM2-8-076-s002.doc]

# Determination of depth and field size dependence of MLC transmission in IMRT beams

Piotr Zygmanski, Florin Rosca, Dnyanesh Kadam, Friedlieb Lorenz,

Adrian Nalichowski, Laurence Court, Lee Chin

*^+^ Department of Radiation Oncology, Brigham and Women’s Hospital and Harvard Medical School, Boston, MA 02115, USA*

[*pzygmanski@LROC.harvard.edu*](mailto:pzygmanski@LROC.harvard.edu)

Treatment of large and complex volumes may result in IMRT plans with relatively large contribution from MLC transmission. In such cases, comprehensive characterization of direct and scatter MLC transmission is important. We designed a set of tests (open beam, closed static multi-leaf collimator or MLC, and dynamic MLC gap) in order to determine dosimetric MLC properties as a function of field size and depth at the central axis.

We developed a generalized model of MLC transmission to account for direct MLC transmission, MLC scatter, beam hardening, and leaf-end transmission (dosimetric gap). The model is consistent with the beam model used in IMRT optimization. We tested the model for extreme asymmetric fields relevant for large targets and split IMRT fields. We applied our MLC scatter estimation formula to clinically relevant cases and showed that MLC scatter is contributing and undesired background dose and is relatively large, especially in low-dose regions (a uniform extra dose may dramatically increase normal lung toxicity in treatment of thorax). For complex IMRT of large-volume targets, direct MLC transmission dose is found to be as high as 30% and MLC scatter up to 10% within the target volume for the selected cases. We identified that the dose discrepancies between IMRT planning system (Eclipse^TM^ (Varian)) and ionization chamber measurements (inside and outside of the field) are due to an inadequate model of MLC transmission in the planning system (constant value model). In this study, we measured MLC transmission properties for Varian 6EX (6MV) and Varian 21EXs (6,10MV); however, the experimental method and theoretical model are more general.

# Introduction

Planning for intensity-modulated radiation therapy (IMRT) and quality assurance (QA) for whole pelvis (e.g. prostate + regional lymph nodes), head & neck, and especially mesothelioma may be challenging due to the complexity of the target (large and difficult geometry often with irregular and concave shapes) and the demanding nature of the optimization objectives (multiple targets with different dose levels, up to a dozen organs at risk). This may lead to very complex and noisy fluence patterns with low monitor unit (MU) efficiency and small average multi-leaf collimator (MLC) gap. MU efficiency of dynamically delivered IMRT fields can be defined as a ratio of the MU needed to deliver an open beam and IMRT for the same field size, depth and average dose per field. MU-efficiency for an open beam would be 100%, and for a completely blocked field (closed MLC) would be equal to the MLC transmission (T≈1.4% for 6MV 2100Ex linac). In our clinical practice, we have encountered a relatively large number of IMRT plans for which the MU efficiency is less than 20%. It is noteworthy that 20% efficiency corresponds to about 81% of the delivered radiation being attenuated and only about 19% being transmitted through MLC gaps. Such low MU-efficiency implies an increased contribution of MLC transmission (mid-leaf, inter-leaf, and tongue & groove), MLC induced beam hardening, and MLC scatter to the total dose.

In addition, because for large targets the field sizes range from about 20 cm x 20 cm to 35 cm x 38 cm, and the target cannot be covered by the range of the MLC motion (14-cm limit on field width), fluence patterns are often split into 2 or 3 sub-fluences. For instance, IMRT with 7-9 fields may result in 12-27 split sub-fields in the delivery.

Dose due to MLC scatter from split fields accumulate in the total plan in the same way for the normal organs and the target but the normal organs may be more sensitive to the extra dose.

Due to cumulative effects, calculation of dose for such fluence patterns is very sensitive to any slight inadequacies of the dose calculation model, and also to the uncertainties in the dosimetric MLC parameters in the IMRT planning system. If an IMRT planning system assumes a constant value model of MLC transmission, this may be inadequate for complex/large target IMRT, for which both the field size and depth dependence of MLC transmission matter.

Dosimetric MLC properties have been studied in the context of dynamic IMRT delivery either by direct measurement and theoretical calculation, or by Monte Carlo simulations^1-7^. To the best of our knowledge, though the problem of MLC scatter and beam hardening in IMRT has been identified^3-5^, it has not been completely characterized and resolved.

The published data on MLC scatter and beam hardening are primarily based on Monte Carlo studies^3,4^. For a closed MLC, the Monte Carlo data indicate a significant field size dependence of the MLC scatter, as well as depth and energy dependence of MLC scatter and beam hardening. For IMRT fields with a high component of closed MLC vs open beam (e.g. 80% closed MLC + 20% open beam) these dependencies are applicable as well. Even though MLC scatter data obtained by techniques other than Monte Carlo studies has been reported, the experimental method that leads to its determination is not fully described^5^.

Even within the Monte Carlo beam model, proper characterization of MLC properties may be difficult, and extensive experimental calibration of MLC parameters used in the Monte Carlo code is required^8,9^. This calibration has to be performed separately for each physical MLC / linac combination. This indicates that Monte Carlo-derived MLC parameters can be used primarily for a Monte Carlo dose calculation engine and that for another dose calculation engine (e.g. planning system) other sets of calibration tests may need to be carried out.

Recently, additional experimental data on the MLC scatter and comparison of dose outputs for dynamic sweeping gaps for two commercial planning systems (Eclipse, Pinnacle for 2300CD and 21EX) were reported^10^. These commercial planning systems do not model the MLC scatter, and the associated errors can be significant^10^.

In this report, we introduce a phenomenological beam model that explicitly includes the MLC scatter. We systematically measure MLC transmission as a function of field size, depth, and beam energy and derive the model parameters. MLC beam hardening is considered only to the extent that it affects MLC parameters, which depend on depth. We provide an approximate formula for estimation of MLC scatter. We test the model for extreme asymmetric fields relevant for large targets and split IMRT fields.

We focus on 6 and 10MV photon energy beams from 6EX (6MV only) and 21EX (6,10MV) Varian linacs equipped with Millenium MLCs (120 leaves, rounded leaf design).

# Materials and methods

## Dosimetric MLC parameters

We introduce a phenomenological method to simultaneously determine dosimetric MLC parameters: direct MLC transmission T, MLC scatter correction β, and dosimetric gap ΔG=(2 RFO+CMO). Radiation Field Offset (RFO) is the offset of MLC positions due to penetration of radiation through the rounded leaf ends, and Center Mechanical Offset (CMO) is a mechanical offset that can be adjusted by the user (typically set to zero)^2,11^. The net effect of the two offsets RFO and CMO is sometimes called a dosimetric gap in Eclipse treatment planning system (TPS) and is equal to ΔG = (2*RFO + CMO).

Except for interleaf transmission, dose profiles of OB, cMLC, and dMLCgap normalized to the central axis are similar. The dynamic MLC tests and analysis presented here were designed to determine the MLC parameters in such a way that the interference of the other effects (extrafocal radiation, interleaf MLC transmission, tongue & groove effect) is either null or negligible. In principle, direct MLC transmission and MLC scatter radiation include both the primary radiation and head scatter. Both the primary and head scatter radiations are attenuated by the MLC.

In our model MLC parameters do not include head scatter, nor phantom scatter, both of which are effectively canceled by determining appropriate dose ratio (same field size and depth). Changes in beam spectrum due to MLC are reflected in depth dependence of T(d) and β(d). The spatial dependency of the MLC transmission due to the tongue & groove MLC design and its impact on complex IMRT delivery will be discussed in more detail in a future report.

OB-cMLC-dMLCgap tests apply to dynamic MLC field sizes from c_x_ × c_y_ ≈ 3x3 to c_x_ × c_y_ ≈14x40 cm^2^. However, because for sizes smaller than c_x_ × c_y_ ≈5x5 cm^2^ MLC scatter is negligible and dosimetric gap is practically independent from field size, it is sufficient to use a minimum field size of about c_x_ × c_y_ ≈ 5x5 cm^2^. Since the field size dependence is smooth, the largest field sizes we use are c_x_ × c_y_ ≈ 12x28 cm^2^ with 30x30 solid water phantom and 14x38 cm^2^ with 30x(2*30) cm^2^ solid water phantom.

## Dose ratios

We limit phenomenological modeling of the experimental data to dose ratios: R_cMLC_, R_dMLCgap_. The ratios express the relationship between doses in test conditions and doses under the reference conditions at the central axis and SDD=100cm. These ratios are derived from the measured doses D_OB_, D_cMLC_, and D_dMLCgap_ for open beam (OB), closed MLC (cMLC), and dynamic MLC gap (dMLCgap) accordingly:

$R_{cMLC}\text{ }=\text{ }\frac{D_{cMLC}\left(c_{x},c_{y},d\right)}{D_{OB}\left(c_{x},c_{y},d\right)}$ (1A)

$R_{dMLCgap}\text{ }=\text{ }\frac{D_{dMLCgap}\left(c_{x},c_{y},d\right)}{D_{OB}\left(c_{x},c_{y},d\right)}$ (1B)

The doses are measured for the same field size and depth (c_x_ × c_y_, d). The ratio R_cMLC_ is associated with direct MLC transmission T(d) and MLC scatter β(d) parameters, and R_dMLCgap_ is associated with dosimetric gap parameter ΔG(d).

## Closed MLC tests

The ratio of closed MLC dose to open beam dose R_cMLC_ has two contributing factors, which are related to direct MLC transmission and MLC scatter. We assume that the primary, head scatter, backscatter, and phantom scatter photons in the closed MLC measurement have properties similar to those in the open beam measurement, except that they are attenuated and scattered by the MLC (except the backscatter from the jaws, which does not reach the MLC). The photons transmitted and scattered by the MLC have an altered spectrum compared to the open beam photons, but this is effectively taken into account in the depth dependence of the MLC parameters, as described below.

According to Monte Carlo simulations^3,4^, MLC scatter radiation is rather uniform across the field. At off-axis point placed at x=7cm MLC scatter contribution decreases by 5-3% lower than on the central axis when normalized to the total transmission at the central axis for 5x5-20x20 fields^3^. MLC scatter at the central axis is shown to be 3-23% of the total MLC transmission for 5x5-20x20 fields^3^. For these reasons, we adopted a uniform model of distribution of MLC scatter. We verify this approximation for off-axis points and for asymmetric fields by performing measurements as described below.

Thus, we adopt the following parameterization of the MLC transmission and MLC scatter

$R_{cMLC}=T\left(d\right)\left(1+\beta\left(d\right)c_{eff}^{2}\right)$ (2A)

$c_{eff}=\frac{\left(1+k\right)c_{x}c_{y}}{k\text{ }c_{x}+c_{y}}$ (2B)

where MLC scatter parameter β(d) is defined as a correction to direct MLC transmission T(d), so that the total MLC transmission is T(d) (1+β(d) c_eff_^2^) and c_eff_^2^ is the area of an effective square field. This definition is similar to the definition of scatter-to-primary ratio (SPR) used in modeling head S_H_ and phantom scatter S_P_ (References 14-18). Effective field size c_eff_ is expressed in terms of collimator exchange factor k (k≈1.3 for Varian 21Ex open beam data, we used k=1.35 for modeling MLC transmission).

The MLC transmission is a function of depth, since for low- and medium-energy linacs there could be a 5-10% increase in PDD due to the MLC-induced beam hardening^3^. We assume a linear depth dependence for direct MLC transmission T(d)

$T\left(d\right)=T\left(d_{\max}\right)\left(1+\kappa\text{ }\left(d-d_{\max}\right)\right)$ (3)

in which κ is a beam hardening parameter. The specific functional form is confirmed by the experimental results. The magnitude of the MLC scatter is expected to diminish with increasing depth due to a difference in spectrum of the directly MLC transmitted photons vs MLC-scattered photons.

## Dynamic MLC gap tests

We have adopted a definition of fluence found in IMRT planning systems^5,11-13^. This definition of fluence is not exactly the physical definition (the number of particles per unit area). For this reason we use a notion of ray density φ, which is a mathematical concept used in IMRT optimization and dose calculation. It is essentially a density of rays in the isocentric plane emanating from a point source at SAD=100cm. The value of φ for the open beam or static MLC is 1 inside the field, and 0 outside the field (with sharp edges, no penumbra).

The dynamic MLC gap dose is a combination of the open beam and closed MLC dose contributions. The open beam contribution is proportional to the direct radiation and is associated with a direct ray density φ in our notation. The closed MLC contribution is proportional to the radiation attenuated by the MLC and is associated with (1-φ) term. In principle, direct ray density φ can take any value between 0 and 1 for dynamic MLC delivery.

The Radiation Field Offset (RFO) and Center Mechanical Offset (CMO) are included in the definition of φ by offsetting all the leaf positions by a constant gap ΔG/2 = RFO + CMO/2. Typically CMO is set to values close to zero. Parameter ΔG is called dosimetric gap in Eclipse^7^. It effectively accounts for the non-uniform leaf-end transmission and remaining mechanical offset. If an MLC dose not have a rounded leaf-end design, then ΔG=CMO. For a dynamic MLC gap of size G, moving at a constant speed across a distance Δx, the direct ray density is uniform and its value is

$\varphi_{dMLCgap}=\frac{G+\Delta G}{\Delta x}$ (4)

Dose ratio R_dMLCgap_ is a sum of the open beam and closed MLC ray densities

$R_{dMLCgap}=\varphi_{dMLCgap}+\left(1-\varphi_{dMLCgap}\right)R_{cMLC}$ (5)

where the first contribution is due to primary fluence through the MLC gaps, and the second term is the transmission/scatter through the MLC.

## Correction of TPS fluence model

*Model of fluence in the treatment planning system*

Let us consider a fluence as defined by the planning system (Eclipse) Φ_TPS_. In the current TPS fluence model, the MLC transmission T is an effective constant for all field sizes and depths. In this model the total fluence is essentially a ray density Φ_TPS_ ^11,12,19^

$\Phi_{TPS}=\varphi+T_{TPS}\left(1-\varphi\right)$ (6)

where φ is a general ray density. The value of φ for the open beam or static MLC is 1 inside the field, and 0 outside the field (with sharp edges, no penumbra).

There are differences between the treatment planning system MLC parameters and the parameters used in our model. The planning system does not allow more than two fixed parameters: (T, ΔG)_TPS_. The model of MLC transmission presented here has three parameters: (T, β, ΔG)_exp_. In both models the parameters are determined from experiment and accordingly they have different values. However, in TPS it is difficult to find one set of parameters (T, ΔG)_TPS_ that will account for all field size, depths, and fluence complexity.

*Correction of TPS model*

It is instructive to evaluate the importance of the MLC scatter for various levels of the effective ray density 〈φ〉. The effective ray density 〈φ〉 is a spatial average of φ within the limits set by the jaws [φ = 1 ⇒ 〈φ〉 ≡ 1, see also References 11 and 12]. In the following discussion we ignore the dependence on depth d of the MLC parameters. If we assume that the MLC scatter is spatially uniform across the field, which is a good first-order approximation according to References 3-5 and our own results, it is possible to modify the treatment planning model of ray density Φ_TPS_ by simply adding an MLC scatter correction term and changing the meaning of the MLC transmission parameters

$\Phi_{new}=\varphi+T_{new}\left(1-\varphi\right)+T_{new}\left(1-\left\langle\varphi\right\rangle\right)\beta_{new}c_{eff}^{2}$ (7)

This approach is clear once we realize that a convolution of the uniform MLC scatter kernel with a ray density φ is equivalent to taking an average of the ray density. These relations are validated by performing central axis and off-axis measurements for sweeping gap and for complex IMRT plans.

*A scaling law*

In clinical practice approximate scaling laws are used for various dosimetric estimations. For this reason we give a crude estimate of MLC scatter in an IMRT field in terms of 〈φ〉. Based on Equation (7), the relative contribution of the MLC scatter for an IMRT field is

$\frac{\left\langle\Phi_{new}\right\rangle-\left\langle\Phi_{new}\right\rangle_{\beta=0}}{\left\langle\Phi_{new}\right\rangle}=\frac{T_{new}\beta_{new}c_{eff}^{2}}{\left\langle\varphi\right\rangle/\left\langle1-\varphi\right\rangle+T_{new}\left(1+\beta_{new}c_{eff}^{2}\right)}$ (8)

The advantage is that it shows how the MLC scatter contribution depends on the complexity of IMRT plan expressed by spatially averaged ray density 〈φ〉 (a “scaling law” in terms of 〈φ〉).

## OB-cMLC-dMLCgap tests

*MLC parameters*

Direct MLC transmission T(d) and MLC scatter β(d) parameters are determined from the Equations (2A) and (2B) and the measured dose ratio R_cMLC_ for various sizes. The dosimetric gap ΔG(d) is determined independently from the other MLC parameters using Equations (4), (5) and the measured dose ratios R_cMLC_ and R_dMLCgap_. We used Levenberg-Marquardt fitting method in Mathematica^TM^ (Wolfram). Experimental methods used in determination of MLC parameters and application of the model of MLC transmission to dynamic IMRT are described below.

*Determination of MLC parameters with OB-cMLC-dMLC tests*

Open beam (OB), closed MLC (cMLC), and dynamic MLC sweeping gap (dMLCgap) tests were performed for a selected combination of rectangular (jaw) field sizes: c_x_ × c_y_ at a given depth d in a solid water phantom at the machine isocenter and for a given beam energy (Figure 1). The dynamic MLC gap (dMLCgap) test was performed for various constant gap sizes G, traveling at a constant speed across a distance Δx=c_x_+G.

Specifically, the following irradiation conditions were applied:

(i) field sizes c_x_ × c_y_ [cm × cm] = 5x5, 7x7, 10x10, 12x12, 12x20, 12x28,

(ii) dMLC gaps G = 0.1, 0.5, 1.0, 2.0cm,

(iii) depths d=d_max_, 5, 10 cm, where d_max_=1.5 cm for 6MV and 2.5cm for 10MV

(iv) 6EX (6MV) and 21EXs (6MV and 10MV) Varian linacs equipped with Millenium MLCs

The measurements were performed at the central axis with an ionization chamber placed at isocenter, perpendicular to the beam and perpendicular to the MLC leaves. We used an A12 (Exradin) ionization chamber (0.6 c^3^ active volume). The detector size was sufficiently large to average out the inter- and intra-leaf transmissions during the measurement. Thus the inter-leaf leakage effect for the cMLC and dMLCgap tests is effectively incorporated into the MLC parameters. The tongue & groove effect is not present in the cMLC or dMLCgap tests (the tongue or groove leaf sides are not exposed to radiation separately).

*Filtering of the scatter with stereotactic cones*

Additional open-beam and closed MLC measurements were performed to separate the MLC scatter signal from the MLC transmission signal in the R_cMLC_. These measurements were performed by placing an ionization chamber with a buildup cap in air and irradiating the detector twice: with and without a stack of collimating cones (stereotactic cones). A stack of lead cones 38cm long with diameters 2.75-3.25cm was placed immediately above the detector. An ionization chamber TDC100 with a water equivalent buildup cap of thickness 1cm was used. Otherwise the setup was the same as in Figure 1.

*MLC transmission at off-center points, inside/outside of field for symmetric/asymmetric fields*

The OB-cMLC-dMLCgap tests as a function of field size and depth were measured for symmetric field sizes (x1=x2=c_x_/2, y1=y2=c_y_/2) at the field center. To test the variation of MLC scatter at off-center points inside/outside of field for symmetric/asymmetric fields additional open beam and closed MLC tests were performed for various asymmetric jaw settings. Jaw sizes were chosen to depict the extreme jaw positions within the limits imposed by dynamic MLC delivery: (x1, x2, y1, y2) [cm, cm, cm, cm] = (2.5, 2.5, 2.5, 2.5), (5, 5, 5, 5), (7, 7, 7, 7), (7, 7, 10, 10), (7, 7, 15, 15), (7, 7, 19, 19) symmetrical fields; (x1, x2, y1, y2) = (7, 7, 15, 19), (7, 7, 11, 19), (7, 7, 7, 19), (7, 7, 3, 19) x-jaws symmetric and y-jaws asymmetric; (x1, x2, y1, y2) = (7, 7, -3, 19), (7, 7, -7, 19), (7, 7, -10, 19), (7, 7, -10, 14), (7, 7, -10, 10) y1-jaw covers the detector and x-jaws symmetric; (x1, x2, y1, y2) = (16, -2, 2.5, 2.5), (16, -2, 5, 5), (16, -2, 7, 7), (16, -2, 9, 10), (16, -2, 15, 15), (16, -2, 19, 19) x2-jaw covers the detector and y-jaws symmetric; (x1, x2, y1, y2) = (16, -2, 15, 19), (16, -2, 11, 19), (16, -2, 7, 19), (16, -2, 3, 19) x2-jaw covers the detector and y-jaws asymmetric; (x1, x2, y1, y2) = (16, -2, -3, 19), (16, -2, -7, 19), (16, -2, -10, 19), (16, -2, -10, 14) x2-jaw and y1-jaw cover the detector.

These measurements were done both inside and outside of the field in air with A12 ionization chamber inside an Aluminum buildup cap of d=1.5cm. Contributions from jaw transmission outside of the field were subtracted from the total ionization chamber signal as described below. Otherwise the setup was similar to that shown in Figure 1.

We compared MLC scatter calculated according to the model described above (uniform MLC transmission in Equations (2A) and (2B)) with the ionization chamber measurements using the following dose ratios as figures of merits. For points measured inside the field a dose ratio R^in^_cMLC_ is sufficient to describe MLC transmission

$R_{cMLC}^{in}=R_{cMLC}^{off}\text{ }$ (9A)

However, for points outside of field there is no direct MLC transmission and there is an extra jaw transmission. The jaw transmission adds an offset signal and is subtracted from the total dose ratio R^off^_cMLC[_ in order to obtain R^out^_cMLC_, which properly characterizes MLC transmission outside the field

(9B)

where the total (auxiliary) dose ratio was determined from the measurements

(9C)

and term is an extrapolation to zero field size for asymmetric jaws. The above dose ratios $R_{cMLC}^{in}$and$R_{cMLC}^{out}$ are exactly the same as R_cMLC_ (defined by Equation (2)) for symmetric fields at the central axis but they are expected to be smaller than R_cMLC_ for asymmetric fields (or for off-center points) due to a gradual fall-off of the MLC scatter with the increasing off-center distance.

*Dynamic MLC gap tests outside of field*

Our model of MLC transmission, strictly speaking, applies to IMRT at the central axis. Field size dependence is shift invariant in our model, which is an approximation. To test the validity of our beam model outside of field, additional open-beam and dynamic MLC gap tests were performed using an ionization chamber for a limited number of cases. Specifically, the ionization chamber was placed at x=0, 7, and 10cm. The field sizes were c_x_ × c_y_ = 10 cm x10cm, 14cm x 30cm. Depth was d=10cm and energy was 6MV. Otherwise the setup was similar to that shown in Figure 1.

*Magnitude of MLC scatter for complex IMRT: central axis*

In order to measure the magnitude and uniformity of MLC scatter for complex IMRT plans, we performed measurements in solid water with an A18 ionization chamber (0.125 c^3^) at depth of 1.5cm at the central axis. The presented IMRT plan consisted of 23 fluences (9 fields requiring 23 split fluences), MU-efficiency of the plan was about 8%, field sizes were large (c_x_≈14cm, cy≈34cm), and treated volume was large (≈30cmx30cmx34cm). Thus the MLC transmission and scatter contributions were sufficiently significant to test the spatial dependence for complex IMRT.

We performed two series of measurements with various magnitudes of total scatter component: one for large y-jaw settings y_1_=y_2_=17cm (cy=34cm) and another for cropped y-jaws y_1_=y_2_=2.5cm (cy=5cm). Calculation of doses was done with uniform MLC scatter component (β>0) and without MLC scatter (β=0) according to Equation (7).

We compared measured to calculated relative IMRT doses: ((D_34cm_-D_5cm_)/D_34cm_)_exp_ vs ((D_34cm_-D_5cm_)/D_34cm_)_β=0_ and ((D_34cm_-D_5cm_)/D_34cm_)_β=β_. The values of MLC parameters (T,β,ΔG) were determined from OB-cMLC-dMLCgap test and MLC scatter dose was calculated according to Equation (2). By taking the respective dose ratios ((D_34cm_-D_5cm_)/D_34cm_), we minimized the effects due to dose gradients. Otherwise the experiment was the same as in Figure 1.

*Magnitude of MLC scatter for complex IMRT: 2D*

In addition, we performed measurements with ionization chamber array (MatriXX, Wellhofer) for a clinically relevant case. Measurements were performed and analyzed at d=15cm for zero gantry angle. An IMRT plan composed of 9 fields was split into 23 sub-fields. The field sizes of the original fields were large and covering about 30cm x 30cm area. We compared the measured doses to TPS doses and our calculations of MLC scatter using Equations (7) and (8). We limited analysis to 24cm x 24cm area (size of detector) and determined total dose per field and total dose for the composite plan within 24cm x 24cm area. Total (or average) dose per field and per plan is a good descriptor of the magnitude of MLC scatter for the following reasons.

Comparison of 2D doses with TPS for clinically relevant cases is possible. However, interpretation requires caution. If a large fluence map is split into 2 or 3 sub-fields, the dose bias is additive in the composite fluence, as well as in the composite plan. Because the MLC scatter is more or less uniform, while IMRT fluences for complex plans are very irregular and show many low-dose regions, analysis of dose differences due to MLC scatter in terms of total dose within the field (or average dose) is preferred. This dose bias is seen more clearly in a composite plan, which is more uniform.

## IMRT planning system

*Calculation of doses using treatment planning system (TPS)*

Experimental OB-cMLC-dMLCgap tests were simulated using an IMRT planning system (Eclipse Version 7.3.1), and doses were calculated for the same setups. Dose points and profiles were exported to compare them to the measured data. The plans were calculated for the dMLC parameters currently used clinically. For instance, TPS parameters used at our department are: T_TPS_ =1.4%, 1.4%, 1.7% and ΔG_TPS_ =0.175, 0.175, 0.190 for the energies considered. Parameter (*β*)_TPS_ is not defined in TPS. Dose rate R=400 MU/min was used. The ray density calculation in the planning system depends on the dose rate because the MLC speed is finite, which must be taken into account.

In the OB-cMLC-dMLCgap test, we consider more parameters: (T, ΔG, β), which depend on depth. These parameters must be considered together. In this sense, it does not make much sense to compare the numerical value of the treatment planning system MLC parameters to the parameters in our model. The doses resulting from an application of the two models (ours vs TPS ) are better quantities for such a comparison.

## Error analysis

In order to see the relation between the uncertainties in the MLC parameters and the relative fluence errors, we consider a general case. Below δ refers to the uncertainty in the determination of the MLC parameters (T, ΔG, β) and ray density Φ.

Generally, the relative ray density error due to the uncertainty δT in the determination of the MLC transmission T can be evaluated from a formula in Reference 7

$\left(\frac{\left\langle\delta\Phi\right\rangle}{\left\langle\Phi\right\rangle}\right)_{T}\cong\delta T\frac{1-\frac{\left\langle G\right\rangle}{\Delta x}}{\frac{\left\langle G\right\rangle}{\Delta x}}\cong\delta T\frac{\Delta x}{\left\langle G\right\rangle}$ (10A)

and the errors due to the uncertainty in the dosimetric gap δ(ΔG)= (2 ΔRFO+CMO) from a formula in Reference 8

$\left(\frac{\left\langle\delta\Phi\right\rangle}{\left\langle\Phi\right\rangle}\right)_{\Delta G}\cong\frac{\delta\left(\Delta G\right)}{\left\langle G\right\rangle}$ (10B)

From the above, if <G> is of the order of 0.5-1.0cm and Δx=10-15cm, even small uncertainties δT=0.001 or δ(ΔG) =0.05cm contribute significantly to the ray density errors. These contributions have even greater importance in the regions outside of the target and within the organs at risk, where the fluence is generally relatively low.

# Results

## Closed MLC tests

An example of R_cMLC_ (closed MLC dose to open-beam dose ratio) as a function of the field area (c_x_×c_y_) is shown in Figure 2. Measurements were made with the cone (▲) and without the cone (🞧) in air to show that the MLC scatter is responsible for the field size dependence of R_cMLC_. By placing a stack of cones above the detector, the MLC scatter was effectively removed.

In addition, Figure 2 shows R_cMLC_ measured without the cone in solid water at d_max_ (⯃) vs R_cMLC_ measured without the cone in air (🞧). These two data sets are the same to within the uncertainty of the measurements, indicating that phantom scatter does not bias the derivation of the MLC parameters, because it is effectively canceled when the dose ratio is taken. Based on the in-air measurements with and without cone, head scatter in open beam is about 4%, and field size dependence of the closed MLC is about 25% for the same for the field sizes considered (5-20cm). Closed MLC dose includes head scatter contribution similar to the open beam dose. However, MLC transmission has only the remaining about 21% field size dependence attributed to scatter from the MLC. This confirms that MLC scatter parameter β does not include head scatter.

Table 1 shows the MLC parameters derived from the OB-cMLC-dMLCgap test for d=d_max_, 5cm, 10cm, and two Varian linacs (6EX and 21EXs). The beam-hardening parameters κ based on Equation (3) are: 0.0075 cm^-1^ (6MV 6EX), 0.010 cm^-1^ (6MV 21EXs), and 0.0027 cm^-1^ (10MV 21EXs). Because the TPS does not model the MLC scatter explicitly, direct comparison of (T, *β*, ΔG) values from the OB-cMLC-dMLCgap test to the planning system values may be misleading.

The numerical results are consistent with Monte Carlo (MC) studies for a similar linac^3^, except that the MC data for 18MV indicate that MLC scatter relative to MLC transmission is lower than for 6MV. Apparently, 10MV is not sufficiently different compared to 6MV to decrease the MLC scatter contribution significantly.

## Dynamic MLC gap tests

The dosimetric gap ΔG is determined from Equations (4) and (5) independently from the transmission and the MLC scatter parameters. The results shown in Figure 3 validate this methodology. Figure 3 shows an example of the measured dose ratio R_dMLCgap_ divided by the right side of Equation (5) derived from measured OB and cMLC data for various gap sizes G and field sizes (c_x_,c_y_) for d=10cm. The data are contained within a ±1% window. This disagreement is due in part to noise and in part to other inherent uncertainties described below. However, it has negligible effect on the model parameters and potential dose errors that may result (see error discussion section below).

## MLC parameters

MLC parameters determined from OB-cMLC-dMLCgap tests are shown in Table 1, together with uncertainties in their determination. These uncertainties include noise in the input data to the fitting programs, and the other systematic uncertainties due to the MLC file itself, choice of detector, and reproducibility. These uncertainties result in the uncertainties of the ray density according to Equations (10A) and (10B):

Δx/G = 10 - 20 and δT =± 0.00025 ⇒ ΔΦ/Φ = ± (0.25% to 0.5%);

G = 0.5cm – 1.0cm and δΔG =± 0.005cm ⇒ ΔΦ/Φ = ± (0.5% to 1.0%);

Δx/G = 10 – 20, c=20cm and δβ =± 0.2x10^-4^cm^-2^ ⇒ ΔΦ/Φ = ± (0.2% to 0.3%).

The uncertainty in T and β add in the formula (2A). MLC misalignment (CMO) is automatically included in ΔG and is not considered a part of the inherent uncertainty of the method.

| **d[cm]** | **T_exp_[1]** | **(β)_exp_ [10^-4^ cm^-2^]** | **ΔG_exp_[cm]** |
| --- | --- | --- | --- |
| **d_max_** | 0.0144 / 0.0139 / 0.0170 | 6.4 / 6.5 / 6.2 | 0.146 / 0.157 / 0.174 |
| **5** | 0.0147 / 0.0144 / 0.0171 | 5.8 / 5.8 / 6.2 | 0.147 / 0.159 / 0.176 |
| **10** | 0.0153 / 0.0151 / 0.0174 | 5.1 / 5.0 / 5.7 | 0.148 / 0.162 / 0.177 |
| **δ(T, β,ΔG)** | δT= ±0.00025 | δβ= ± 0.2x10^-4^cm^-2^ | δ(ΔG) = ± 0.005cm |

**Table 1**

Parameters (T, *β*, ΔG) determined from the OB-cMLC-dMLCgap test as a function of depth for different machines and energies. The three numbers in each field refer to the three machines: 6MV 6EX linac, 6MV 21EXs, and 10MV 21EXs.

## Non-uniformity of MLC scatter

Figure 4 shows field size dependence of calculated R_cMLC_ according to Equations (9A,9B,9C) and measured $R_{cMLC}^{in}$,$R_{cMLC}^{out}$ dose ratios. As expected, R_cMLC_ is a little bit larger than $R_{cMLC}^{in}$and$R_{cMLC}^{out}$ for all combinations of jaw sizes. However, the field size dependence is stronger than the dependence on the distance to field center, indicating that a model of uniform MLC scatter gives a good estimate (an upper limit) of MLC scatter.

## Dose profiles for dynamic MLC gap patterns

A comparison of the TPS dose profiles and profiles corrected to account for the MLC scatter for the dynamic MLC gap test vs measurement (three points) is shown in Figure 5. Correction due to MLC scatter was derived from Equations (4) and (5) and was applied the same way at the central axis and at off-axis points inside and outside of the field according to Equation (7). The corrections in absolute doses outside the field are comparable to the differences at the central axis. This is because MLC scatter is relatively uniform; points outside the field receive almost the same amount of MLC scattered radiation. As expected based on Equations (4) and (5), the MLC scatter effect is stronger for smaller gaps and larger field sizes. Experimental validation of the transmission model for arbitrary IMRT fluence patterns is described below.

## Magnitude of the MLC scatter for clinical cases

*Dose difference (c_y_ =34cm vs 5cm) with and without MLC scatter*

Figure 6 shows experimental and calculated dose differences: ((D_34cm_-D_5cm_)/D_34cm_)_exp_ vs ((D_34cm_-D_5cm_)/D_34cm_)_β=0_ and ((D_34cm_-D_5cm_)/D_34cm_)_β=β_. The total measured doses for a cumulative plan were about 110cGy (c_y_ =34cm) and 85cGy (c_y_ =5cm). The calculated doses due to direct MLC transmission were about 25%(34cm), 30%(5cm), and due to MLC scatter 12%(34cm), 2%(5cm). It can be seen that MLC scatter is especially large for low-dose fields (ionization chamber outside of a specific sub-field). In fact, MLC scatter is about inversely proportional to the total dose, and is more or less equal to the total head scatter dose plus phantom scatter dose from regions outside of c_y_ =5cm at d=1.5cm.

*2D verifications*

We compared the measured MatriXX doses to TPS doses and our calculations of MLC scatter. Measured fluence maps were found to be very similar in shape to TPS maps, however, a small dose bias was observed. Total dose bias per field was ranging from (+6.4%) to (+9.1% ) and was (+7.5%) for the composite plan dose. Our calculation of the magnitude of MLC scatter according to Equations (7) and (8) resulted in very similar numbers ((+4.2%) to (+10.5%) per field and (+7.8%) for composite plan). This shows that the discrepancy between the TPS and measurement is predominantly due to MLC scatter. The small discrepancies between our estimation of MLC scatter vs experimentally determined dose biases for individual fields may be due to the dose gradients (averaging effect of finite size detectors) and presence of T&G effects.

*Scaling law*

In routine IMRT QA for less complex cases, these sort of effects are difficult to demonstrate in a single fluence with an ionization chamber (insufficiency of data points and the problem of gradient), or with 2D detector (problem of film normalization). The cumulative measurement, including all the fields with an ionization chamber, should reveal this sort of discrepancy, but not necessarily its nature (MLC scatter origin).

For this reason we present an approximate scaling law formula (Equation (8)) describing how to calculate effective fluence error as a function of average fluence level, which may be helpful in predicting the MLC scatter effect on IMRT plans of various complexity. Plan complexity, dose output efficiency, and the effective MLC gap size are all related^11,12^.

Figure 7 shows the relative magnitude of MLC scatter as a function of 〈φ〉. For small field sizes (c=5cm, 10cm) the magnitude of MLC scatter is small, but for the larger sizes (c=20cm) it is only small when 〈φ〉 >0.3. 〈φ〉 =0.3 implies that about 3 times (1/0.3) more monitor units is used for IMRT delivery compared to monitor units for an open beam for the same depth and field size. In practice, 〈φ〉 can be as small as 0.1, for complex IMRT plans. If the field size is large for these cases, the magnitude is significant.

# Discussion

*General*

In the commissioning of an IMRT planning system, the OB-cMLC-dMLCgap tests can be used for a smaller number of field sizes (e.g. 5x5, 10x10, 12x28) and MLC gaps (e.g. 0.1cm, 1.0cm) than were used in this study and at one clinically relevant depth. This amounts to about 3x3x2=18 measurements.

Our study is complementary to previous reports^3,4,10^. In our method, in addition to the sweeping gap (dMLCgap) and open beam (OB) tests, we performed closed MLC (cMLC) tests and described MLC properties in terms of 3 MLC parameters (different figures of merit) derived from the measured data: ratios of closed MLC dose to open beam dose and ratios of sweeping gap dose to open beam dose. Based on published Monte Carlo simulations^3^, for high-beam energy (15MV), the relative amount of MLC scatter to MLC transmission decreases considerably. However, as our study shows, the amount for 10MV it is almost the same as for 6MV.

More studies are needed to understand the impact of field size and depth dependence of total MLC transmission for real clinical cases, especially on normal organs, that are often in the low-fluence areas (and therefore the relative dose error might be larger than for the target). The confounding factors are the nonuniformity of the fluences and the fact that they are split unevenly (into 2 or 3 sections, with the overlap in various places), patient anatomy (relative position of the target with respect to the normal organs), and the location of inhomogeneities. Equation (7) is still valid for clinical cases to the degree that the MLC scatter is uniform and can be represented by 〈φ〉. A more accurate description would involve using (x,y)-dependent MLC scatter in a manner similar to that used in References 14-18.

Small out-of-field dose errors for the open beams or static MLC conformal plans due to inadequate penumbra modeling (different from MLC aperture effects discussed in this report) may not be clinically significant. However, in complex IMRT with split-fluences, these small errors may be magnified and/or add up to significant dose errors, especially to the normal organs, as our study shows. For these reasons, it is recommended that for complex IMRT plans (low 〈φ〉) doses outside of the isocenter and outside of the target be measured as well (for instance, with several films placed in various slices in a phantom).

We suggest that an ideal IMRT planning system should include MLC scatter parameters and enable the user to easily commission the system in the beam configuration, using the concepts described above for the cMLC and dMLCgap (or similar). It is conceivable that the standard beam data (PDD/TPR, S_p_, S_BC_, S_H_, OAR, etc), which forms the core of the TPS, be extended to include the MLC scatter factor S_MLC_, in a similar way as the collimator backscatter S_BS_ and head scatter S_H_. In severely complex IMRT, it may be important to include the depth dependence of the transmission and the depth dependence of the MLC scatter, though it would be more difficult to implement this in the dose calculation algorithm in the presence of inhomogeneities.

We have not performed measurements for other MLC designs. However, we expect that our model would still apply to other MLC designs and linacs with minor modifications. The model parameters would change their values. For instance, for MLC without rounded leaf-ends, ΔG=0. The situation here is similar to modeling head scatter. Further, if the MLC scatter becomes more pronounced and /or more dependent on off-axis distance than for Varian MLC, there could be a need to introduce a Gaussian model of MLC scatter distribution resulting in a β(d) Erf(c/λ)^2^ correction [17] instead of β(d) c^2^, which we used in our model. However, it would have to be determined experimentally if these modifications are necessary.

*Choice of optimal TPS parameters and potential dose errors*

The TPS has only two parameters (T, ΔG)_TPS_, while more parameters are needed: (T, β, ΔG)_exp_ to more fully characterize dosimetric MLC properties, which depend of field size and depth. It is not possible to find one set of parameters (T, ΔG)_TPS_, which would work for all field sizes and all depths. Thus, one has to find such a set of parameters (T, ΔG)_TPS_ that minimize the dose errors due to the MLC scatter. One option is to create multiple machines with different values of (T, ΔG)_TPS_ in TPS: one for small field IMRT (c_x_ × c_y_ ≤ 10×10), and another for large-field IMRT (c_x_ × c_y_ ≥ 14×20). However, this may not be practical.

Figure 8 shows an approximate dose errors for a situation in which the effective TPS parameters (T, ΔG)_TPS_ are determined for 10×10 (according to Equation (6)) and used in the TPS for dose calculation for other field sizes, neglecting the MLC scatter effect. Gap sizes are G=0.5cm, 1.0cm, 2.0cm and the dose error is considered in the high-dose region. In such case, the dose discrepancy for the field sizes above about 15x15 is quite large. Interpretation of Figure (8) needs caution.

It is the large field size and low MU-efficiency that lead to significant dose error, if field size dependence of MLC transmission is neglected. Each split field contributes to the in-field and out-of-field regions more or less the same amount of the undesired MLC scatter dose. One may be under an impression that because the split fields have lower field sizes than the original fields (e.g. three 14cmx35cm fields instead of one 35cm x 35cm), MLC scatter effect is less significant in the total plan. This is not true, because what matters is the field size of the original field before splitting, which tends to be large for large targets.

For these reasons in Figure 8 three additional curves are shown. The curves denoted by (x 3) refer to an extreme case when a large field is split into three sub-fields. Because the errors from individual sub-fields add up, the total error is up to three times larger. Since the actual MLC scatter decreases slightly with the off-axis distance (Figure 4), errors in Figure 8 show the upper limit for dose errors due to MLC scatter only.

Another crucial point is that for split fields the extra dose impacts the target and normal organ regions, but normal organs are more sensitive to the same amount of extra dose than the target. Figure (8) shows relative dose error in the high-dose region (target region). Relative dose errors in low-dose regions are considerably larger. Since for complex IMRT optimization is pushed to the limits and often normal organs receive maximum allowed dose, this is makes the dose bias even more significant.

*Other MLC effects*

Analysis of the OB-cMLC-dMLCgap test presented here assumes that the detector is sufficiently large that the inter- and intra-leaf transmissions are averaged during the measurement. Because all the leaves of a sweeping gap are moving in the same fashion, there is no tongue-and-groove effect in the OB-cMLC-dMLCgap tests. The tongue-and-groove effect would be present if the leaf sides of individual leaves were exposed to radiation. However, for delivery of IMRT to the patient, : (i) the inter- vs intra-leaf transmission, and (ii) the tongue-and-groove effect are enhanced when MU-efficiency is low and the leaf sides are often exposed during the leaf motion. Both of these effects result in an undesired modification of the ideal fluence. While (i) is effectively included in the notion of the spatially averaged MLC transmission T (Equation (2)), (ii) may cause an overall underdosage of the target, which can be numerically estimated only if the tongue and/or groove MLC transmissions are directly included in the fluence calculations. This negative dose bias may be significant when fluence delivery is complex^20^. Furthermore, even though the usage of non-coplanar IMRT fields and/or varying collimator angle can blur the effects of (i) as well as (ii), it cannot alter the total amount of dose deposited within the target due to (ii). This aspect of complex IMRT delivery will be the focus of a future study.

We have assumed that MLC backscatter into the monitor chamber is negligible. The reason for this is that the backscatter from the upper jaw is not more than 2% for Varian linacs^6^ and therefore it should be significantly less for the MLC, and in fact it is below the uncertainty of our experiment (1%). Should the MLC backscatter play a more significant role, we would have to include a new correction factor with (-α_MLC,BS_) parameter in Equation (3).

# Conclusions

Ignoring field size dependence of MLC transmission may lead to underestimation of dose inside and outside of the field in treatment of large targets. The exact amount of MLC scatter dose depends on the total field size (before splitting) and on MU-efficiency, which can be described in terms of effective ray density <φ>. For sweeping gap tests used in the report <φ> = (G+ΔG) / (c+G). The undesired dose bias due to MLC scatter from split fields (inside and outside of the split field) add up in the total plan. MLC scatter for clinically relevant IMRT plans are found to be up to about 10% of the total dose within the target and more outside of the target. The latter may be more important, for instance, in treatment of thorax because of sensitivity of lung to low-dose when large lung volumes are irradiated.

We have presented a phenomenological model of beam transmitted through MLC and provided MLC tests (OB-cMLC-dMLCgap) to be used to determine the model parameters. The model can be used in commissioning of treatment planning system or in developing of new dose calculation algorithms that would directly model MLC scatter and beam hardening. The inclusion of these additional parameters improves the accuracy of dose estimation, if the dose calculation algorithm is modified according to our model.

# Acknowledgements

We would like to express our gratitude to Mike Makrigiorgos for offering us dosimetry support.

# References

1. Boyer A, Biggs P, Galvin J, Klein Eric, LoSasso T, Low D, Mah K, Yu C, AAPM Report No 72, ‘Basic applications of multileaf collimators: Report of the AAPM radiation therapy committee Task Group No. 50’ (2001)
2. LoSasso T, Chui CS, Ling CC, ‘Physical and dosimetric aspects of a multileaf collimation system used in the dynamic mode for implementing intensity modulated radiotherapy’, Med Phys 25, 1919-1927 (1999)
3. Kim JO, Siebers JV, Keal PJ, Arnfield MR, Mohan R, ‘A Monte Carlo study of radiation transport through multileaf collimators’ Med Phys 28, 2491-2505 (2001)
4. Arnfield MR, Siebers JV, Kim JO, Wu Q, Keal PJ, Mohan R ‘A method for determining multileaf collimator transmission and scatter for dynamic intensity modulated raditherapy’ Med Phys 27, 2231-2241 (2000)
5. Chui CS, LoSasso T, Palm A ‘Computational algorithms for independent verification of IMRT’ in ‘A practical guide to Intenisty-Modulated Radiation Therapy’, Medical Physics Publishing, Madison Wisconsin (2003)
6. Jiang S, Boyer A, Ma CM ‘Modeling the extrafocal radiation and monitor chamber backscatter for photon beam dose calculation’ Med Phys 28, 55-66 (2001)
7. Zhu T, Ahnesjo A ‘Interpretation of in-air output ratio and its impact on dose calculation’ AAPM Refresher Course, July 2004
8. MC Pönisch F, Titt U, Kry SF, Vassiliev ON, Mohan R, ‘MCNPX simulation of a multileaf collimator’, Med Phys 33, 402-404 (2006)
9. Jang SY, Vassiliev ON, Liu HH, Mohan R, Siebers JV, ‘Development and commissioning of a multileaf collimator model in Monte Carlo dose calculations for intensity-modulated radiation therapy’, Med Phys 33, 770-781 (2006)
10. Higgins PD, Alaei P, ‘Dose uncertainty due to aperture effects in dynamic fields’, Med Phys 33, 2418-2425 (2006)
11. Zygmanski P, Kung JH, 'Method of Identifying Dynamic Multileaf Collimator Irradiation that is Highly Sensitive to a Systematic MLC Calibration Error ', Med Phys 28(11), 2220-2226 (2001)
12. Zygmanski P, Hacker F, Friesen S, Rodenbush R, Lu HM, Chin L, ‘Maximum MLC opening effect in dynamic delivery of IMRT: leaf-positional analysis’, J Clinical Med Phys 6, 33-43 (2005)
13. Webb S, ‘Intensity-Modulated Radiation Therapy’, Institute of Physics, Series in Medical Physics (2001)
14. Dong L, Shiu A, Tung S, Hogstrom K ‘A pencil beam photon dose algorithm for stereotactc radiosurgery using a miniature multileaf collimator’ Med Phys 25, 841-850 (1998)
15. Bjarngaard BE, Vadash P, Ceberg AP ‘Quality control of measured x-ray beam data’ Med Phys 24 1441-1444 (2003)
16. Zhu T, Bjarngaard BE, Xiao Y, Yang CJ ‘Modeling the output ratio in air for megavoltage photon beams’ Med Phys 28 925-937 (2003)
17. Zhu T, Bjarngaard BE ‘Head scatter off-axis for megavoltage x-rays’ Med Phys 30 533-543 (2003)
18. Zhu T, Bjarngaard BE ‘Output ratio in air for MLC shaped irregular fields’ Med Phys 31 2490-5433 (2004)
19. Eclipse Manual, Varian, Palo Alto, 2004
20. Que W, Kung J, Dai J**'Tongue-and-groove' effect in intensity modulated radiotherapy with static multileaf collimator fields’** Phys Med Biol **49**, 399-405 (2004)

**Table 1**

Parameters (T, *β*, ΔG) determined from the OB-cMLC-dMLCgap test as a function of depth for different machines and energies. The three numbers in each field refer to the three machines: 6MV 6EX linac, 6MV 21EXs, and 10MV 21EXs.

**Figure 1**

Schematic diagram of the OB-cMLC-dMLCgap tests. The depth d, field size c_x_ × c_y_ are the same for all three cases. The detector is placed at the isocenter perpendicularly to the beam. From the above measurements R_cMLC_ and R_dMLCgap_ dose ratios are determined.

**Figure 2**

An example of the measured (data points) and fitted (straight lines) dose ratios R_cMLC_ with and without a cone for 6MV 6EX linac. Measurements were made in air with the cone (▲), in air without the cone (🞧), and in solid water at d_max_ without the cone (⯃). Ionization chambers A12 and TDC100 were used. The discrepancy between the two sets of data without the cone (🞧 and ⯃) is within the accuracy of the method.

**Figure 3**

Example of the measured dose ratio R_dMLCgap_ divided by the right side of the Equation (5) (“formula”) for four gap sizes G and six field sizes (c_x_,c_y_) for d=10cm. “Formula” is equal to ( φ_dMLCgap_+(1-φ_dMLCgap_)R_cMLC_ ) and is derived from measurements and Equation (4). Derivation of the dosimetric gap ΔG is based on Equation (5). Dosimetric gap is practically constant for various field sizes and depths as shown in Table 1.

**Figure 4**

Measured vs calculated total MLC transmission (direct and scatter) for various x- and y-jaw positions as a function of field area. Doses were measured for Varian 6EX (6MV) in air with A12 ionization chamber and Aluminum buildup cap. For the points outside of the field (x2), (y1) and (x2, y1) indicate the jaw(s) behind which the ionization chamber was placed.

**Figure 5**

Absolute dose profiles [cGy] for the TPS and ionization chamber measurements (points) for an extreme sweeping gap of 0.6 mm width, for two field sizes: 10cmx10cm and 14cmx30cm. Also shown are corrected profiles utilizing Equations (4), (5) and (7).

**Figure 6**

((D_34cm_-D_5cm_)/D_34cm_) vs absolute dose D_34cm_ per field determined by measurement, calculation without MLC scatter and calculation with MCL scatter. D_34cm_ signifies dose per field for an IMRT plan with c_y_=34cm, and D_5cm_ dose for the same plan with y-jaw cropped to 5cm.

**Figure 7**

A relative magnitude of MLC scatter based on Equation (8) as a function of the effective ray density 〈φ〉 for square jaw sizes: 5cm (solid), 10cm (long dashed), 20cm (dotted). Parameters used: T=0.015 and β=0.0006.

**Figure 8**

Magnitude of dose errors due to MLC scatter as a function of square field size c when the effective TPS parameters (T, ΔG)_TPS_ are determined for 10×10 (according to Equations (6)) and dose is calculated by the TPS for other field sizes c without the additional MLC scatter correction. Gap sizes G=0.5 cm (solid), 1.0cm (dashed), 2.0cm (dotted). Sweeping gaps travel distances of (c+G). (x 3) denotes the total error when a larger field is split into 3 smaller fields. Field size c is the size of a split sub-field.
